# Omicron-included mutation-induced changes in epitopes of SARS-CoV-2 spike protein and effectiveness assessments of current antibodies

**DOI:** 10.1186/s43556-022-00074-3

**Published:** 2022-04-24

**Authors:** Du Guo, Huaichuan Duan, Yan Cheng, Yueteng Wang, Jianping Hu, Hubing Shi

**Affiliations:** 1 Laboratory of Tumor Targeted and Immune Therapy, Clinical Research Center for Breast, State Key Laboratory of Biotherapy, West China Hospital, Sichuan University and Collaborative Innovation Center, Chengdu, 610041 China; 2 Key Laboratory of Medicinal and Edible Plants Resources Development of Sichuan Education Department, School of Pharmacy, Chengdu University, Chengdu, 610106 China

**Keywords:** SARS-CoV-2, Spike protein, Epitopes, Antibody, Drug resistance

## Abstract

The COVID-19 pandemic, caused by severe acute respiratory syndrome coronavirus 2 (SARS-CoV-2), is spreading globally and continues to rage, posing a serious threat to human health and life quality. Antibody therapy and vaccines both have shown great efficacy in the prevention and treatment of COVID-19, whose development progress and adaptation range have attracted wide attention. However, with the emergence of variant strains of SARS-CoV-2, the neutralization activity of therapeutic or vaccine-induced antibodies may be reduced, requiring long-term virus monitoring and drug upgrade in response to its evolution. In this paper, conformational changes including continuous epitopes (CPs), discontinuous epitopes (DPs) and recognition interfaces of the three representative SARS-CoV-2 spike protein (SP) mutants (i.e., the Delta (B.1.617.2), Mu (B.1.621) and Omicron (B.1.1.529) strains), were analyzed to evaluate the effectiveness of current mainstream antibodies. The results showed that the conformation of SP wild type (WT) and mutants both remained stable, while the local antigenic epitopes underwent significant changes. Sufficient flexibility of SP CPs is critical for effective antibody recognition. The DPs of Delta, Mu and Omicron variants have showed stronger binding to human angiotensin converting enzyme-2 (hACE2) than WT; the possible drug resistance mechanisms of antibodies against three different epitopes (i.e., NTD_DP, RBD1_DP and RBD2_DP) were also proposed, respectively; the RBD2 of Delta, NTD of Mu, NTD and RBD2 of Omicron are deserve more attention in the subsequent design of next-generation vaccines. The simulation results not only revealed structural characteristics of SP antigenic epitopes, but also provided guidance for antibody modification, vaccine design and effectiveness evaluation.

**Supplementary Information:**

The online version contains supplementary material available at 10.1186/s43556-022-00074-3.

## Introduction

Since the emergence of severe acute respiratory syndrome coronavirus 2 (SARS-CoV-2), [1] the COVID-19 pandemic has swept the world posing a serious threat to public health and economic development [2]. Moreover, the high variability of SARS-CoV-2 makes it more difficult for illness prevention and pandemic control [3]. By 16 November 2021, this outbreak has caused more than 253 million cases worldwide [4]. Many SARS-CoV-2 variants, including Alpha (B.1.1.7), Beta (B.1.351), Gamma (P.1), Delta (B.1.617.2), Mu (B.1.621) and Omicron (B.1.1.529), showed increased infectivity and virulence [5]. Supplementary Table 1 lists these strains of concern: up to August 2021, the Delta variant was the dominant strain with a higher transmission rate; [6, 7] the Mu variant has the highest levels of resistance to serum-mediated neutralization [8], which also need special concern; According to the emergency meeting of the World Health Organization (WHO) held on 26 November 2021, the Omicron variant has more mutations than the Delta and is even considered the most dangerous coronavirus strain. The epidemic has been going on for more than two years with no end in sight [9]. Routing prevention and control strategies, including quarantine, face mask, sterilization and clinical therapies (such as respiratory and circulatory supporting therapy, nutrition support and analgesics-sedatives), need to be maintained.

**Fig. 1:**
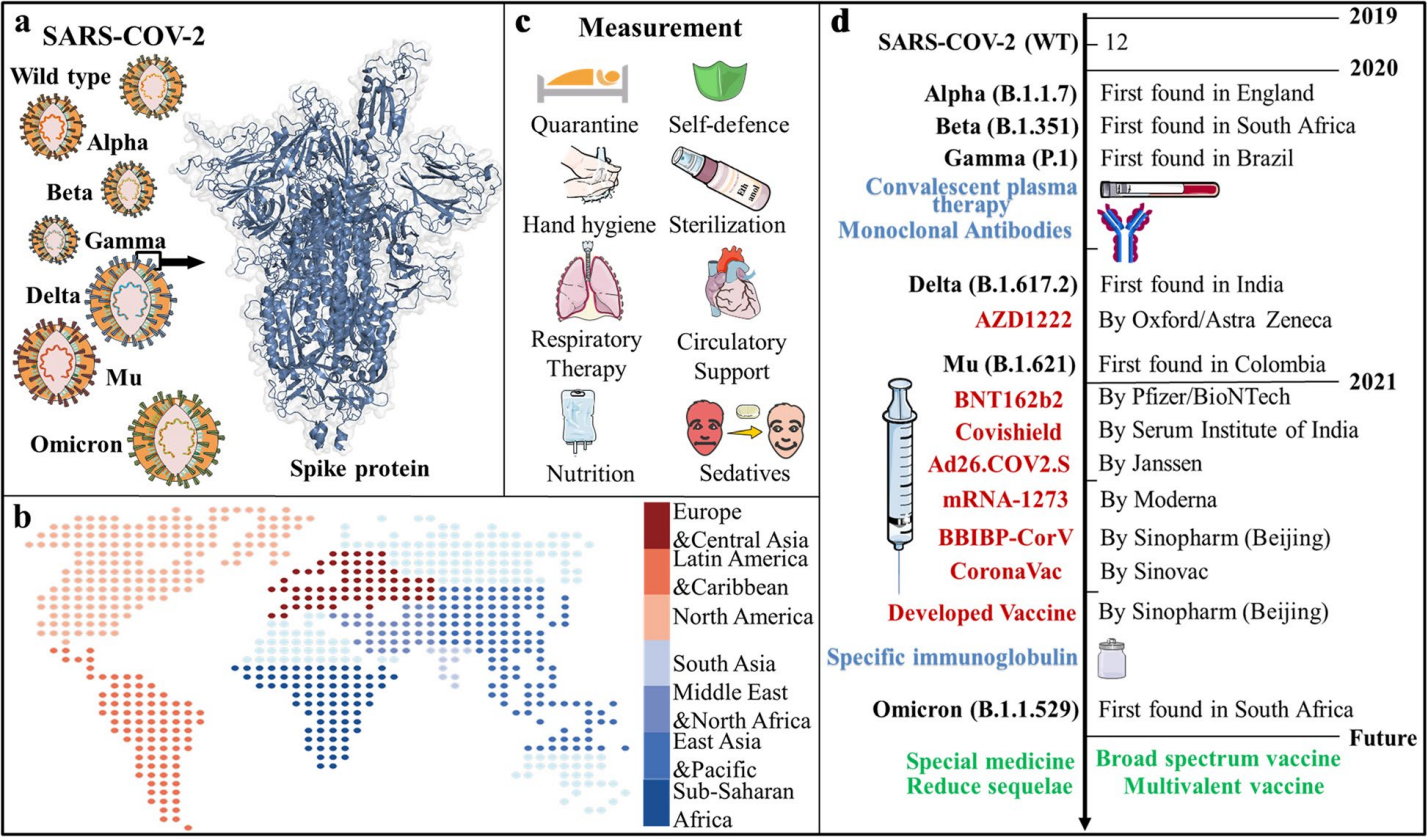
Distribution and treatment strategies of COVID-19. **a** Schematic overview of the SARS-CoV-2 spike protein trimer; **b** cumulative distribution of confirmed COVID-19 cases worldwide by region (The darker the red, the more people are infected with COVID-19, the darker the blue, the less people are infected); **c** measures to deal with COVID-19; **d** a time line of SARS-CoV-2 variants with treatments in blue, vaccines under Emergency Use Authorization (EUA) in red and expectations in green

The vaccines for SARS-CoV-2 are widely administered around the world, [10] with more than 7.5 billion injections till November 16, 2021. Currently, there are 184 / 104 candidate vaccines in preclinical clinical development [11]. COVID-19 vaccines mainly include nucleic acid, viral vector and subunit-based types according to preparation process,and they respectively target the full-length SARS-CoV-2 spike protein (SP) or its receptor binding domain (RBD). Disturbingly, the vaccines appear to become less effective against SARS-CoV-2 variant strain. From February to October 2021, three types of vaccines from America have showed significantly declined effectiveness against infection with the Delta variant rising to dominance, [12] although protection against hospitalization and death still remained high [13]. Other novel therapies such as convalescent plasma, neutralizing monoclonal antibodies, specific immunoglobulin and small molecule drugs have been gradually used and are still under improvement. The use of convalescent plasma owns mortality benefit for patients with COVID-19 according to clinical studies [14]. Some neutralizing monoclonal antibodies (mAbs) have been proved to reduce hospitalizations and mortality with potential utility in prevention of COVID-19 [15]. In addition, the anti-SARS-CoV-2 intravenous immunoglobulin is still under development [16] (see Fig. 1).

As a new therapeutic method, antibody therapy has attracted much attention, although its effectiveness against variant strains remains to be further confirmed [17]. Antibodies to SARS-CoV-2 currently approved by the US food and drug administration (FDA) for therapeutic emergency include [18]: (a) AstraZeneca's Evusheld, a combination of Tixagevimab and Cilgavimab, targeting non-overlapping epitopes of the RBD, (b) Roche's mAbs Actemra, also known as Tocilizumab, targeting IL-6R, (c) GlaxoSmithKline's Sotrovimab binding to a conserved epitope within the RBD, (d) the combination of Bamlanivimab and Etesevimab developed by TopAlliance Biosciences targeting distinct but overlapping epitopes of the RBD, (e) Regeneron's REGEN-CoV targeting the RBD. Given its critical role in the SARS-CoV-2 infection, SP has become a vulnerable target for therapeutic antibody development. In addition to antibody therapy, FDA also recently granted Pfizer Emergency Use Authorization (EUA) for the first oral anti-SARS-CoV-2 drug Paxlovid—a 3C-likeprotease inhibitor—for the treatment of patients with mild to moderate COVID-19.

As one of the essential proteins for the propagation and transmission of SARS-CoV-2 infection, SP mediates viral fusion into cell membranes by interacting with human angiotensin-converting enzyme 2 (hACE2) [19]. SP is composed of two functional subunits, S1 and S2: the former includes N-terminal domain (NTD) and RBD; the latter mainly includes fusion peptide (FP), heptad repeat 1 (HR1), heptad repeat 2 (HR2) and central helix (CH) [20]. After binding with hACE2, SP was preactivated by furin convertase and subsequently cleaved into S1 and S2 parts [21]. The cleavage site located within the S2 subdomain was then exposed and underwent a dramatic conformational change through excision by transmembrane protease serine 2 (TMPRSS2), facilitating membrane fusion [22]. The structure of continuous epitopes (CPs) and discontinuous epitopes (DPs) is crucial for the successful design of therapeutic antibodies; the conformational changes of antigen epitopes not only affect the recognition of ligands, but also determine the effectiveness of induced antibodies [23]. By binding to SP CPs and DPs, some neutralizing antibodies block molecular recognition with hACE2 receptor and partially prevent their incorporation into host cells, thereby weakening the transmission of SARS-CoV-2 [24]. Obviously, comparative studies on epitope characteristic changes between SARS-CoV-2 WT and variants have important guiding significance for the development of next-generation antibodies and vaccines.

The important factors that determine viral infectivity and virulence include SP’s stability, binding affinity to hACE2 and TMPRSS2, as well as SP’s transmembrane capacity, which should be taken into consideration in the following design of highly effective therapeutic antibodies. Currently, the efficacy of neutralizing antibodies against SARS-CoV-2 variant is decreasing, while the reasons remain unclear. In SP mutants, how does the structural change of antigen epitope influence the binding to therapeutic antibodies and thus induce resistance? In the face of numerous variant strains, how to select effective vaccines and how to design next-generation therapeutic antibodies? According to theoretical prediction, what other new mutants may appear in the future? Aiming at the above important scientific issues, this paper mainly consists of two parts: (1) Conformational changes of CPs / DPs in SP WT and three mutants were compared, the difference in recognition with substrates such as antibodies, hACE2 and TMPRSS2 were analyzed; (2) the effectiveness of existing therapeutic antibodies was assessed to aid the design of next-generation vaccines.

## Results

### Characteristics and prevalence of SARS-CoV-2 variant strains

During the current COVID-19 pandemic, SARS-CoV-2 strain has diversified considerably. With the continuous spread of the virus, there have been many strong dominant mutations (such as Delta, Mu, Omicron variants, etc.), which greatly changed infectivity and pathogenicity of virus strain, and attracted the attention of governments around the world.

The Delta variant (B.1.617.2), first detected in India in May 2021, has been designated as a variant of concern (VOC) by WHO [25]. This high-profile variant remains dominant in many regions with more infectivity over other variants [26]. And the Delta variant appeared slightly more prone to immune evasion with reduced sensitivity to neutralizing antibodies from recovered individuals or vaccine induction [27].

The Mu variant (B.1.621), first isolated on January 11, 2021 in Colombia, [28] has been classified as a new variant of interest (VOI) by WHO. So far the B.1.621 lineage is predominantly found in Colombia, the United States, Spain, the Netherlands and Denmark [29]. In addition to other VOC characteristics (e.g., E484K, N501Y, P681H), this lineage SP also showed some new mutations (e.g., R346K, Y144T, Y145S, and 146 N insertion) [30]. The Mu variant is highly resistant to sera from COVID-19 convalescents and BNT162b2-vaccinated individuals, presenting a greater risk of virus spread [31].

A new highly mutated coronavirus variant was first reported to WHO from South Africa designated as the Omicron (B.1.1.529) VOC. As of 29 November 2021, Omicron has been detected in 116 countries. Based on the genome sequences of SARS-CoV-2 isolates recently submitted to CoVariants and GISAID, the Omicron variant has become the predominant strain in South Africa within a month of its emergence, overtaking Delta [32]. Those contracted with the Delta variant have a 40% risk of re-infection with Omicron, according to the studies by Discovery Health, South Africa’s largest insurer [33]. There are up to 43 amino acids mutations in Omicron (see Supplementary Table 1), [34] not only containing common mutations (E484K, N501Y, P681H) reported to reduce neutralization efficacy of antibodies, but also carrying some changes previously not present in other VOCs [35]. It is speculated that excessive mutations may change the behavior of SARS-CoV-2 with regards to immune escape, transmissibility, and susceptibility to some treatments [36].

### Structural stability

The potential energies of WT, Delta and Mu variants maintain stable around at -1.61 × 10^6^ kcal·mol^−1^ with standard deviation of 1.12 × 10^4^, 0.81 × 10^4^, 0.82 × 10^4^ kcal·mol^−1^, respectively; while the Omicron variant is more stable with mean potential energy of -1.68 × 10^6^ kcal·mol^−1^ and standard deviation of 1.11 × 10^4^ kcal·mol^−1^. As shown from Supplementary Fig. 1, the WT, Mu and Omicron variants tend to be stable after 30 ns with root mean square deviation (RMSD) of 4.61, 4.84 and 5.15 Å, respectively, whereas Delta doesn’t reach equilibrium until 65 ns with that of 6.75 Å. It indicates that the three variants are less conformational stable than WT, especially Delta. In addition, the time-dependent RMSD trend of RBD subdomain is similar to that of full-length SP trimer; The average RMSD values of NTD for WT/ Delta/ Mu/ Omicron was 4.22/ 4.26/ 3.88/ 3.72 Å, which are much higher than that of either S2 or RBD alone. It can be speculated that there is a large inter-domain movement between S2 and RBD, which increases conformational displacement of the whole SP.

To analyze the influence of different temperatures on structural stability of SP, comparative MD simulations at three different temperatures (i.e., 300, 310 and 320 K) were conducted. Supplementary Fig. 2 shows trajectory convergence parameters—RMSD, radius of gyration (Rg), root mean square fluctuation (RMSF) and flexibility correlation—for the four SP trimers. The average RMSD values at 300/ 310/ 320 K for WT were 4.61/ 5.47/ 5.05 Å, with those for Delta, Mu and Omicron of 6.75/ 5.34/ 5.24, 4.84/ 4.59/ 5.09, and 5.15/ 4.8/ 4.26 Å, respectively. Rg can be used to describe the overall shape and compactness of the system. The average Rg for the four SP trimers at three temperatures are generally stable, and fluctuate in a narrow range from 49.38 to 51.66 Å. Given that atom number of the simulated systems exceeds 26,000, it can be seen that temperature has little effect on conformational stability of SARS-CoV-2 SP, which is consistent with the previous experimental data [37]. This partly explains why rising temperatures have not significantly attenuated the spread of COVID-19.

RMSF can provide flexibility difference of the simulation system at residual level; the highly correlated RMSF distribution in similar systems can prove the reliability of MD trajectories. As shown in Supplementary Fig. 2, the S2 subdomain has low flexibility except C590-V610 and F800-D830, which is related to multiple helices in this region [38]. The NTD (S13-F318) and RBD (R319-N540) subdomains both are more flexible, especially in the random coil of N440 to C480 from hACE2 receptor binding motif (RBM, N437-P507). According to Yu’s work, [39] the high flexibility of NTD contributes to the recognition of NTD-targeted antibodies, while the greater rigidity of V320-V395 in RBD is conducive to antibodies binding such as CR3022. At different temperatures (e.g., 300, 310, 320 K), the distribution of high flexibility regions of the four SP systems (i.e., WT, Delta, Mu, Omicron) was almost the same, indicating again the little effect of temperature on the overall conformation of the protein. There was a significant correlation of RMSF between Delta/Mu/Omicron variant and WT, with determination coefficient (R^2^) of 0.653/ 0.720/ 0.700, respectively, which fully proved reliability of MD trajectories.

### Conformational fluctuation

**Fig. 2:**
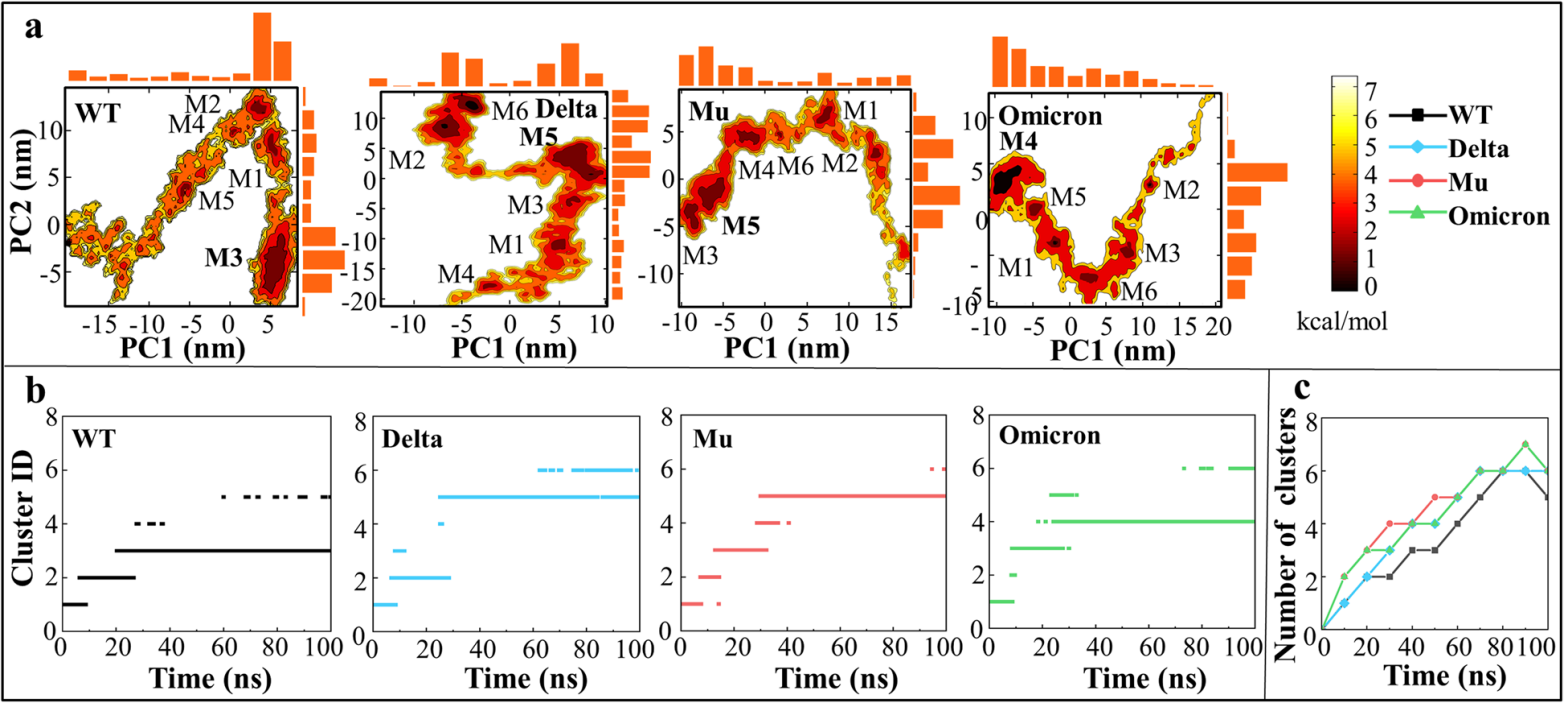
**a** Free energy landscapes of the WT, Delta, Mu, Omicron systems, **b** the corresponding conformational cluster analyses as well as **c** the number of clusters over simulations time. The darker the color in FEL diagram, the lower the conformational free energy

Figure 2 shows free energy landscapes (FEL) and conformational clusters of the WT, Delta, Mu and Omicron systems at 300 K. Along with two dimensional reaction coordinates (PC1 and PC2), FEL are used to describe fluctuation range of representative conformations of the system. The darker the color in the FEL diagram, the more conformations there are, and the lower free energy of the system. According to Fig. 2a, there are five independent low free energy regions in WT, while six in the Delta, Mu and Omicron variants, initially indicating that SARS-CoV-2 SP mutants have greater overall conformational flexibility. Notably, the basin scope of Delta is the largest of all systems showing greater conformational flexibility, which is consistent with the previous RMSD results (see Supplementary Fig. 2).

In order to further analyze the change of the structure with low free energy over simulation time, the conformational cluster analysis was conducted with the RMSD threshold of 3.0 Å. According to Fig. 2b, there are five clusters for WT and six for the other three variants, all of which represent a relatively independent conformational structure, which is completely consistent with the low free energy region obtained by FEL analysis. As shown from Fig. 2c, the time-evolution number of clusters increased gradually in the early stage (0-70 ns), and then declined or kept stable in the late stage (70-100 ns). It indicates that the basic ergodic conformations were fully sampled, providing a solid foundation for subsequent epitope analysis.

### Continuous B-Cell epitopes

**Fig. 3:**
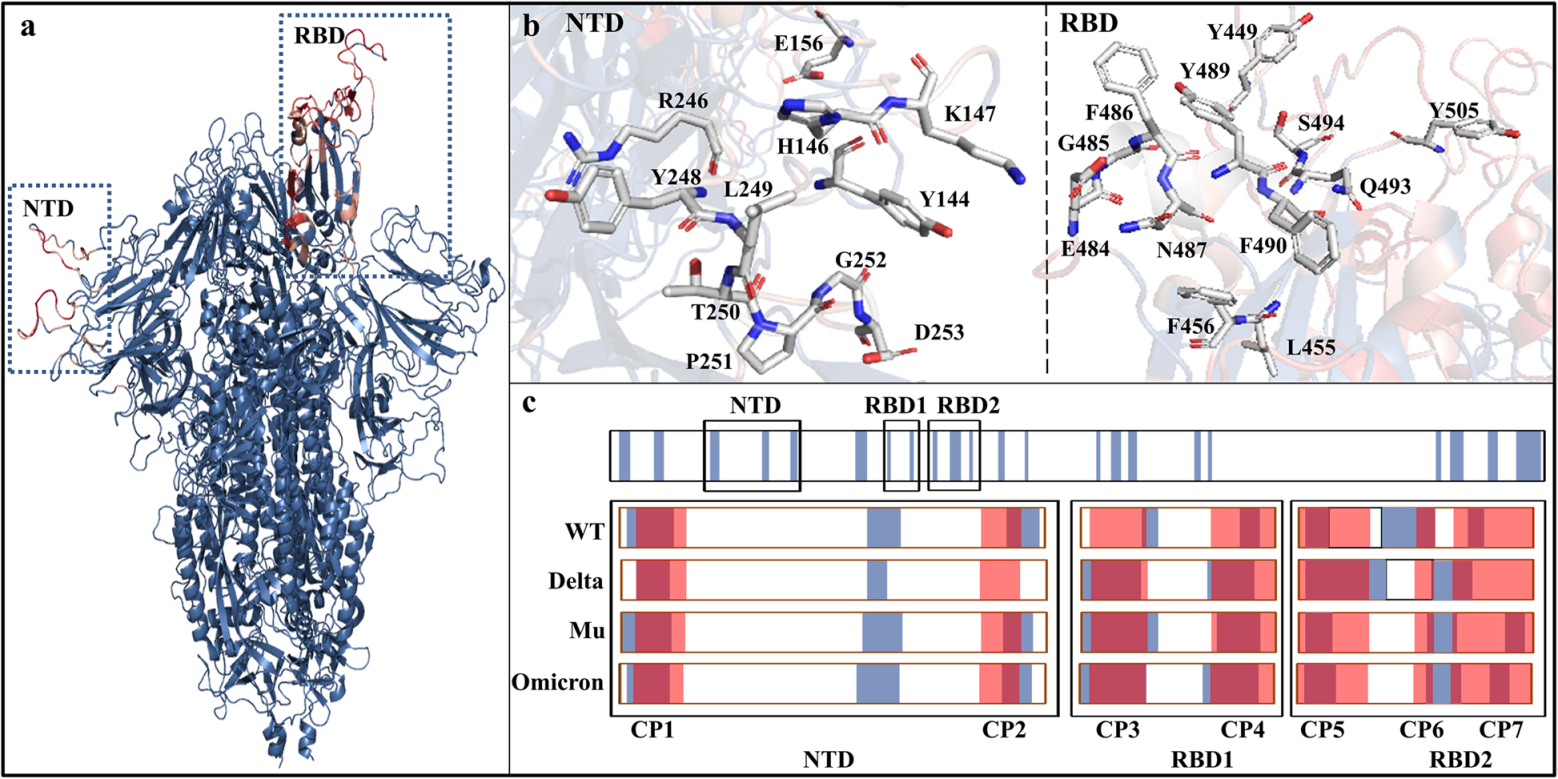
Continuous epitopes analysis of SP. **a** In SP complexes with antibody, the depth of red color indicates the occurrence probability in the interaction residues between SP and antibodies; **b** the possible CPs with occurrence probability over 35% from structural biology statistics; **c** the prediction result with BepiPred-2.0 are colored in blue, the statistical data form RCSB PDB are colored in red, and the overlapped part is the final determined CPs (CP1-CP7)

Figure 3 shows the distribution of predicted continuous epitopes (CPs). Based on the statistics of RCSB PDB structures, all the CPs are located on the surfaces of SP trimer (see Fig. 3a). The high occurrence frequency of residues present in SP-antibody PDB complexes suggests that these regions may be potential CPs, shown in Fig. 3c colored in red. Bepipred-2.0 is a widely used sequence-based epitope prediction tool by training epitopes from antibody-antigen complex structures with random forest algorithm [49]. By Bepipred-2.0 prediction and structural biology statistics, the final continuous B-cell epitopes (i.e., CP1-CP7) were obtained, in the range of Y144-W152, T250-S256, Y369-T385, P412-K417, L441-G447, Y473-S477, G496-N501, respectively. Among the seven SP CPs, CPs 3-4 were localized at RBD1 and CPs 5-7 at RBD2. It should be added that RBD is mainly composed of RBD1 (Y369-D420) and RBD2 (N440-Y505) subdomains [40]: the former provides a conserved implicit epitope for antibodies, that do not overlap with the contact residue to hACE2; the latter not only binds directly with hACE2 to initiate viral entry into cells, but also binds to antibodies at the same site.

In order to compare CPs conformational changes in SP WT and mutants, the representative conformations of each system were superimposed. As shown in Supplementary Fig. 3, CPs 1-2 located at NTD showed large conformational changes, which was consistent with the RMSF analysis above. Indeed, the RMSF values of CPs 1-2 in the Delta, Mu and Omicron variants were lower than that in WT, and the greater rigidity was not conducive to recognition with NTD-targeted antibodies, partly explaining its drug resistance. In addition, the RBD2 CPs in the SP mutants exhibit higher flexibility than WT, which aids the recognition by hACE2 receptor and thus shows higher infectivity and virulence. The flexibility of RBD1 CPs 3-4 was smaller than that of NTD CPs 1-2 and RBD2 CPs 5-7, indicating little difference in binding with RBD1-targeted antibodies, which has some implications for antibody selection and modification.

### Discontinuous B-Cell epitopes

In combination with the EPCES server (scores over 85) and SP-antibody complex statistics, a total of 36 discontinuous epitopes (DPs) residues were identified. These residues were mainly distributed within NTD_DP, RBD1_DP and RBD2_DP regions, with the range of Y144-P251, S375-D420 and G446-Y505, respectively. It is noteworthy that RBD1_DP and RBD2_DP contain 29 residues, accounting for over 80% of the total number of DPs residues. RBD2 contains 22 DPs residues with high immunogenicity, and has become the most important target for SARS-CoV-2 antibody development.

As one of the most important non-bond interactions, H-bond maintains the stability of antigenic epitopes. Supplementary Fig. 4 lists the H-bonds in the WT, Delta, Mu and Omicron systems. The H-bond is defined by geometric criterion: [41] the distance between the donor (D) and acceptor atoms (A) is less than 3.5 Å, and the angle D-H-A is more than 135°. Compared with WT, there was no significant difference in the total number of H-bonds for the other three variants, while their H-bonds with occupancy over 70% are slightly smaller. It indicates that the overall structure becomes relatively loose after mutation, especially for the Mu variant. The Mu variant possesses the most intramolecular DPs H-bonds, nearly twice as the other three systems (i.e., WT, Delta and Omicron), with its epitopes being particularly rigid. Antibodies can form strong H-bond interactions with R457, Q498 and A475, which is critical for maintaining good efficacy [42]. For the Mu variant, too rigid DPs are not conductive to specific recognition of antibodies, and thus more likely to induce antibody escape. Since Y144S is introduced into NTD of Mu, it is speculated that the unique S144-H146 H-bond may be responsible for the rigidity of S144 epitope and the decrease of NTD_DP surface area mentioned later. For the most-concerned Omicron variant, its DPs are relatively unstable in comparison to Delta and Mu. According to Chen’s prediction, Omicron showed significant mAbs-resistance despite its greater CPs flexibility, indicating a new immune escape mechanism [43].

**Fig. 4:**
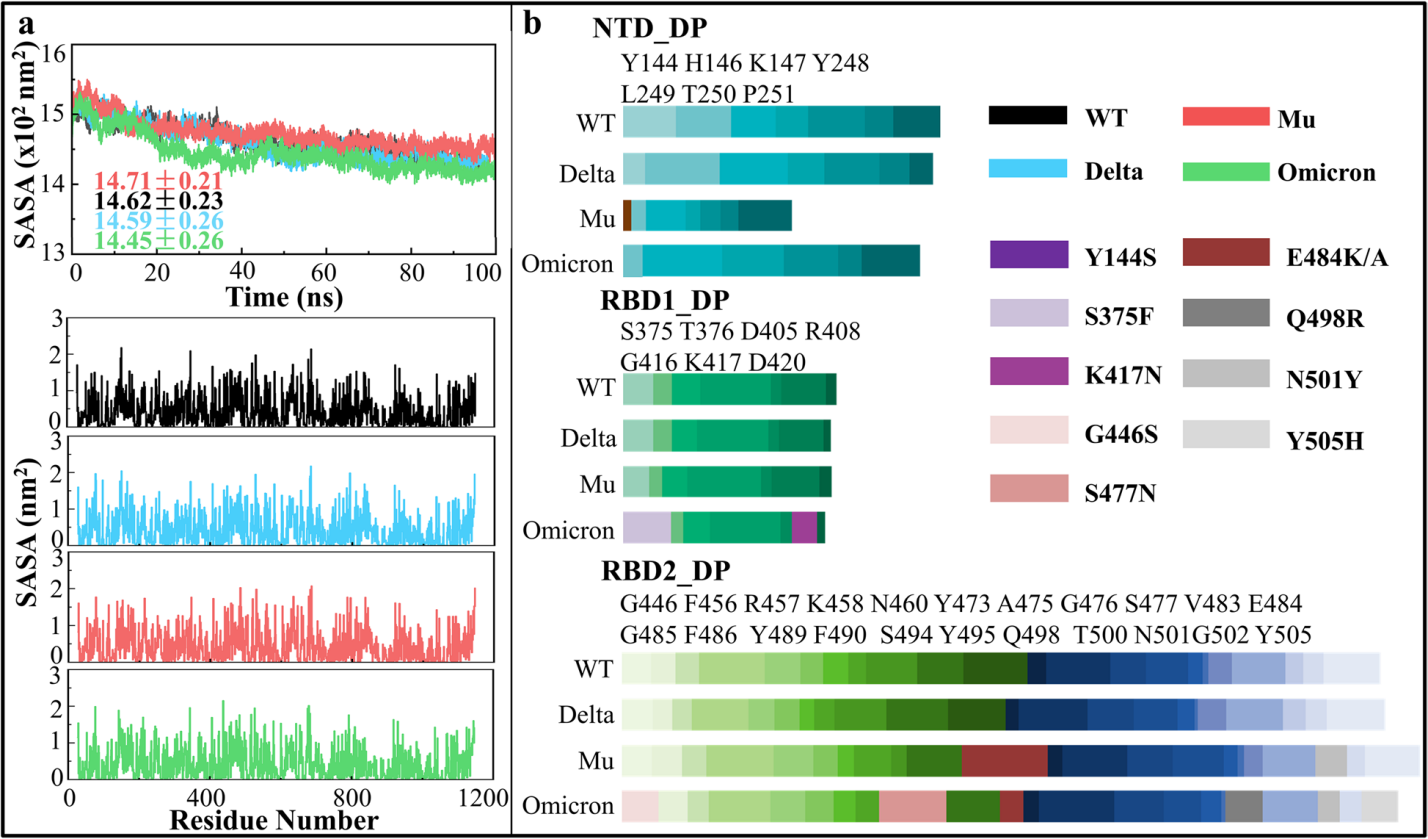
The SASA analyses of the WT, Delta, Mu and Omicron systems. **a** SASA changes at the overall and residual levels; **b** domain-specific SASA for DPs in the NTD, RBD1 and RBD2 subdomains

Figure 4 shows the solvent accessible surface area (SASA) of the WT, Delta, Mu and Omicron systems. As shown from Fig. 4a, the four systems gradually reached a stable state over simulation time; the SASA value of Mu was highest, suggesting that the overall conformation is looser which is consistent with previous H-bond analysis. the SASA distribution trend at the level of residues was roughly the same for the four systems. Comparing the domain-specific SASA of DPs can be used to assess the binding strength of SP to antibodies or hACE2 receptor. As shown in Fig. 4b, the SASA of NTD_DP in the Mu variant was significantly reduced due to Y144S and Y145N mutations. There is no significant difference in RBD1_DP SASA among the four systems; the RBD2_DP SASA of the three variants was slightly higher than WT, which may result in stronger binding ability to hACE2 receptor and increased infectivity of SARS-CoV-2. In conclusion, the SASA of SP variants slightly decreased with different magnitude in NTD, RBD1 and RBD2 subdomains, providing enlightenment for subsequent antibody selection and modification.

Supplementary Fig. 5 shows volume and surface area (SA) of DPs in the WT, Delta, Mu and Omicron systems, which can be used to describe spacial conformation and binding potency with antibody. For the NTD_DPs, the average SAs of the Delta, Mu and Omicron variants are smaller than WT, not conducive to NTD-targeted antibody binding. In the Delta variant, the volume of NTD_DPs was significantly reduced due to the deletion of F157/ R158 in the adjacent loop. The absence of epitope Y144 within the NTD_DPs of Omicron causes SA to shrink and unexpectedly increases in volume. Obviously, this curved surface is not conductive to antibody binding. The average RBD1_DPs volumes of the WT, Delta, Mu and Omicron systems are very close, which is basically consistent with the previous SASA analysis. It will be mentioned later that SARS-CoV-2 variant strains may have special drug resistance mechanism against RBD1-antibody. The increased RBD2_DP SA contributed to the association with hACE2 in all variants, especially for Mu and Omicron that had smaller volume and larger SA (i.e., flatter epitopes).

The charge distribution on antigen surface is critical for antibody recognition and transmembrane transport. Supplementary Fig. 6 shows the surface electrostatic potential (ESP) and total pKa values of each DPs in the WT, Delta, Mu and Omicron systems. The surface of the four systems are alkaline as a whole; all DPs of the variants had higher alkalinity than WT, which was characterized by the sum of pKa value from the dissociated amino acids. The surface electrostatic potential significantly affects the preference of "up" and "down" conformation in RBDs; the alkalinity will contribute to the tendency of “up” conformations to interact with hACE2. Zhou and coworkers [44] revealed that the region N824-L858 can mediate the position of RBD through pH-dependent structural rearrangement: when pH is lower than 5.5, RBD mainly exhibits the "down" conformation. According to Liu and coworkers, [45] a little increase in alkalinity favors the interactions between RBD and hACE2. As shown from Supplementary Fig. 6, the surface ESP of RBD2_DP in Mu and Omicron fluctuated greatly, which was related to the mutations of E484K and Q498R, respectively. In Fratev's research, [46] the K417N mutation could abolish the interactions of SP with STE90-C11 antibody. Obviously, it is necessary to fully consider the surface ESP factor of different SARS-CoV-2 variants in the design of RBD2-targeted antibody.

### The up and down conformations of RBD

**Fig. 5:**
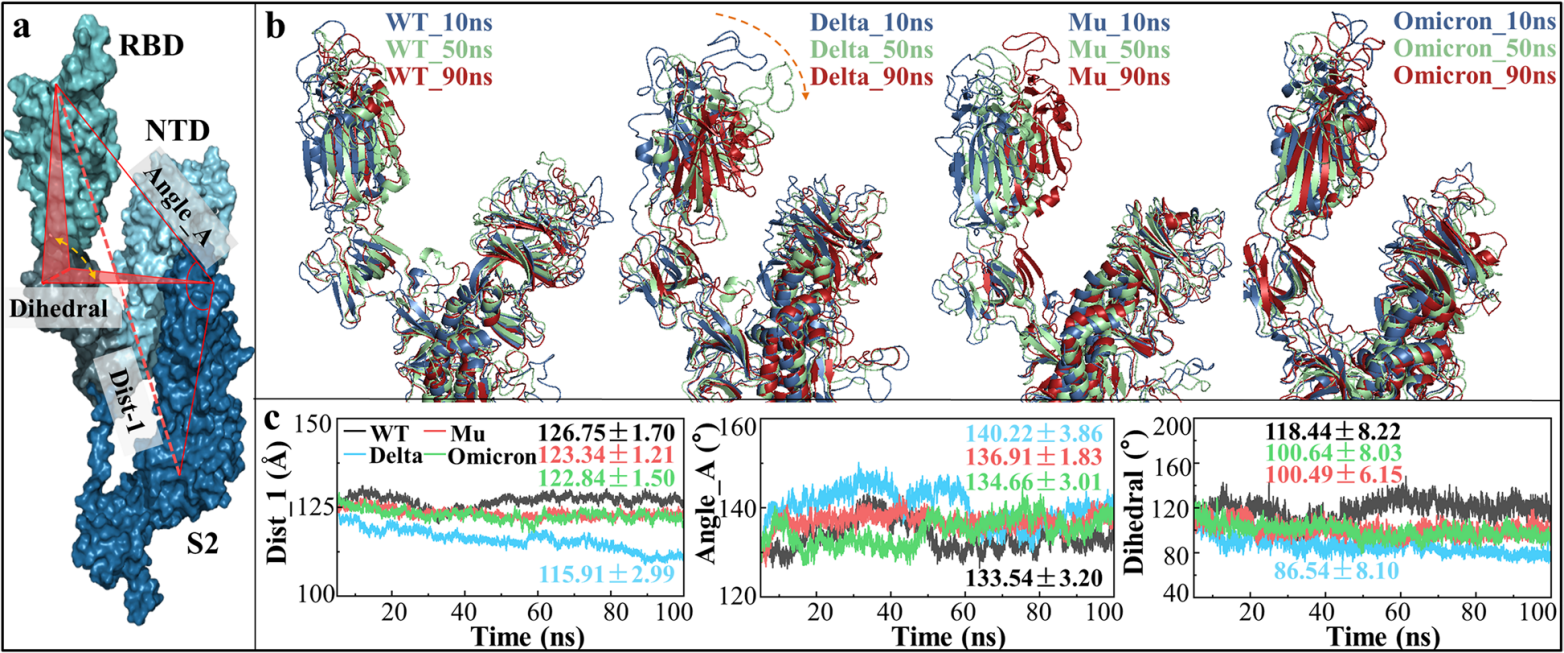
The up and down conformations of RBD. **a** Three parameters are used to describe the relative position of RBD and S2 in SP: the distance (*Dist_1*) between Q493-L1034 Ca atoms, the angle (*Angle_A*) among Q493-K986-L1034 Ca atoms, the dihedral angle (*Dihedral*) among Q493-F543-V576-K986 Ca atoms; **b** structural superimposition from the snapshots at 10, 50 and 90 ns in the four systems; **c** the changes of the three parameters over time

The up and down conformations of RBD corresponds to the active and inactive states of SP, in which the RBD-up state can bind to hACE2 receptor on cell surface guiding the fusion of SARS-CoV-2 into host cells [47]. The question of great interest is whether mutations affect conformational transition between RBD-up and RBD-down states. In addition, the previous RMSF analyses (see Supplementary Fig. 1) have suggested there might be a potential inter-domain movement between RBD and S2 domains. Here, three parameters (i.e., *Dist_1*, *Angle_A* and *Dihedral*) were constructed to describe SP functional conformation and RBD-S2 inter-domain motion (see Fig. 5). Compared with WT, the parameters *Dist_1* and *Dihedral* in the three variants gradually decrease, while the *Angle_A* tends to increase abnormally representing obvious inter-domain movement among RBD, NTD and S2. By superimposing snapshots at 10, 50 and 90 ns, RBD obviously moves towards S2 with an upward tendency during the simulation. It means that the three variants, especially Delta, have a higher probability of staying in the RBD-up state, which agrees well with the previous research results that the D614G mutant tend to be RBD-up conformation [48]. On account of mutation, RBD was significantly closer to S2 and central axis, and its inner side became steeper, making RBD-targeted mAbs harder to approach. Interestingly, the inner side of RBD is exactly the region that RBD1_DP refers to in our work. Given that the overall movement of RBD1-DP, this explains why the previous analysis (including H-bonds, SASA, volume and SA) did not show significant differences in the four systems. The inaccessibility to RBD inside is one of key factors for the resistance of RBD1-targeted antibodies, and the Delta variant may be more likely to escape antibodies with greater steric hindrance.

### Evolutionary conservation

The evolutionary conservation of DPs was analyzed based on their primary sequence, which can be used to evaluate the current dominant mutation characteristics and predict the subsequent possibility mutation selection. Supplementary Table 3 lists the conservation of DPs residues for SARS-CoV-2 SP. Here, the sites with a conservative score less than 3 were identified as weak conservative residues, with high mutational probability. These weak spots are all mainly distributed in the loop of NTD_DPs and RBD2_DPs. Especially, the proportion of weak conservative residues in NTD (residue S13-F318), RBD2 (residue N437-P507) and RBD1 (residue R319-W436) was respectively 58.0%, 60.9% and 21.2%, which indicates higher mutation possibility in the first two regions under natural selection pressure. In NTD, as a high probability mutation residue, Y144 was deleted in the Alpha and Omicron variants, as well as the substitution by S144 in the Mu variant. With the continuous spread of mutated viruses, in addition to Y144, the other 5 weak conservative residues (i.e., H146, K147, Y248, L249 and T250) still have great mutational probability. As for RBD2_DPs, the substitutions of G446S (Omicron), S477N (Omicron), E484K (Alpha, Gamma and Mu), E484A (Omicron), Q498R (Omicron) and N501Y (Alpha, Beta, Gamma, Mu and Omicron) have demonstrated that the weak conservative residues are more susceptible to mutation. The high variation of residues indicates the potential of resistance to NTD- and RBD2-targeted antibodies, and it is necessary to prepare specific vaccines against different SARS-CoV-2 variants.

Although existing vaccines are effective and the number of people vaccinated has increased, they are not enough to end the pandemic [49]. The E484K and N501Y mutants have resulted in reduced neutralization for mAbs, the sera from vaccinator or convalescent patients [50, 51]. In response to reduced protection from existing vaccines, intensive research is underway on the next-generation products against SARS-CoV-2 variants. For instance, there are Moderna’s COVID-19 vaccine (mRNA-1273.351) specially targeted at the Beta variant (B.1.351) [52] and Gritstone’s novel vaccine, which is in phase I clinical trial against additional mutated viral antigens [53]. It is worth mentioning that RBD1_DPs’ conservation is different from NTD_DPs and RBD2_DPs, and only D405 and K417 epitopes are defined as weak conservative spots. K417 has been replaced by Asn in the Beta and Omicron variants, and by Thr in the Omicron variant, leaving D405 with a high mutation possibility in future viral variants. As an important reference target for vaccine development, RBD1_DPs remain higher evolutionary conservation, and RBD1-targeted mAbs may be a universal therapeutic way for different viral variants.

In order to predict possible new mutation sites in subsequent SARS-CoV-2 variants, we calculated total number of DPs residues substituted, H-Bond, SASA, volume, and polarity in six major mutations (see Table 1). Since the highly conservative mutations are the key reason for increased viral infectivity, only the mutated DPs with conservation score greater than 4 were included in further discussion. Through analysis of 15 selected mutations (i.e., E156G, R190S, S371L, S373P, S375F, Y505H, N679K, N764K, D796Y, N856K, D950N, Q954H, N969K, L981F, S982A), it is helpful to explain molecular mechanism of enhanced virulence and attenuated neutralization of therapeutic antibodies in mutants.

**Table 1:**
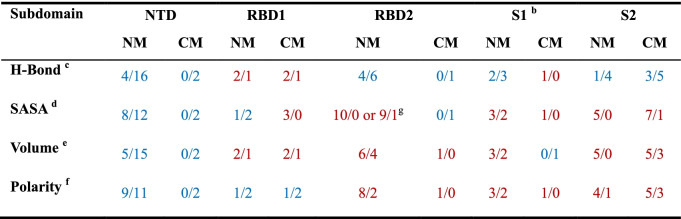
The fluctuation of physicochemical parameters caused by six dominant mutations^a^

^a^ Six dominant mutations include five VOC variants (Alpha, Beta, Gamma, Delta and Omicron) and one VOI variant (Mu); the symbol "/" is preceded by the total number of DP residues with an increase in physicochemical parameters, followed by that with a decrease in parameters; when the number of residues with rising physicochemical parameters is dominant, it is shown in red, otherwise in blue; NM and CM are abbreviations for non-conserved and conserved mutations, respectively

^b^ S1 refers to the portion of full-length SP other than NTD and RBD

^c^ changes of H-bond frequency

^d^ changes of SASA

^e^ change of DP’s volume

^f^ change of residue polarity

^g^ 10/0 is determined from E484A mutation, with 9/1 from E484K mutation

As for the NTD conservative mutations (i.e., E156G and R190S), H-bonds are weakened, SASA and volume become smaller, and polarity decreases, all of which lead to weaker spatial proximity of NTD-targeted antibodies. By altering the local conformation of NTD loops, E156G reduced the neutralization efficacy thus promoting viral infectivity, and R190S impaired the binding ability to antibodies [54 , 55]. E156G is spatially close to the deleted Y144/ Y145 (Alpha) and F157/ R158 (Delta), so it can be inferred that H146, M153 and E154 have a high possibility of deletion or replacement based on conformation proximity and low conservation. Inspired by R190S, H207 on the adjacent β-sheet and E96/ N99 on the adjacent loop may be deleted or replaced in subsequent new viral mutants.

Only the Omicron variant has the unusual mutations including S371L, S373P and S375F in the RBD1 domain, which is a cryptic site hard to expose [56]. The introduction of larger side-chain mutations to RBD1 will increase steric hindrance, thus adversely affecting antibody recruitment. It is consistent with the previous experimental data: the S371L/ S373P/ S375F mutations could reduce affinity to a subset of neutralizing antibodies such as CR3022 and S304 [57]. Referring to Supplementary Table 1 and 3, it can be predicted that E340, K356, S366, N388, S399, R403, G404 and D405, which are less conservative, may be replaced by larger residues with less polarity in the subsequent new viral mutants.

In the Omicron variant, Y505H in RBD2 facilitates the formation of alkaline environment and promotes the association with hACE2. Previous studies have shown that Y505H leads to the loss of interactions between RBD and antibodies [58]. Considering the tendency of increasing volume and polarity for introduced residues in RBD2 (see Table 1), there may be potential substitutions at sites L441, S443, N450, L455, S459, E471, G476, G482, G485, T500, G502, V503 and G504. In combination with evolutionary conservation and statistical changes of physicochemical parameters, it can also be inferred that the possible mutations in S1 (F541-S686, namely the junction between RBD and S2) include T547, N556, P561, I569, T604, L629, P631, T632, T638, S640 and A684. Due to conformational rearrangement of S2 (V687-G1273) from pre-fusion to post-fusion states, the prediction of substitution sites in this region for subsequent new SARS-CoV-2 variants is relatively uncertain. Kumar et al. predicted N764K in the Omicron variant may impair protein function using I‐Mutant 3.0 tool [59]. The results of artificial intelligence (AI) show that Q954H may affect SP fusion state with potential enhanced infectivity and transmission of the Omicron variant [60].

### Effect of SP mutation on virus infectivity

SARS-CoV-2 completes its replication and transmission, relying on the binding of SP to host cells [61]. To provide more information on antibody modification and vaccine design, several key steps in cellular entry process were discussed, including host cells identification via hACE2 binding, cleavage at S1/S2 site by TMPRSS2 and cell–cell fusion by the key subdomain HR1 [22].

As the first step of viral infections, the high affinity between SP and hACE2 is conductive to its higher transmission rate [62]. Based on the trajectories of four comparative 100 ns MD simulations for SP_hACE2 complex models, the binding free energy of WT_hACE2 is -11.24 kcal·mol^−1^ agreeing well with the experimental data [3]. It proves the reliability of prediction method, and the same parameters were also applied to binding free energy calculations between SP and TMPRSS2. As shown from Table 2, the electrostatic energy (*ELE* + *PBELE*) is more negative than hydrophobic energy (*VDW* + *PBSUR*), suggesting that electrostatic energy may be the main driving force to the recognition of hACE2 by SP. The calculated binding free energies of Delta/ Mu/ Omicron SP trimers with hACE2 are -17.31/ -59.12/ -55.22 kcal·mol^−1^, which are obviously stronger than that of WT_hACE2, supporting higher transmission rate [63 , 64].

**Table 2:** The calculated binding free energies of the eight SP complexes (kcal·mol^−1^)

Systems	*ELE*^a^	*VDW*^b^	*PBELE*^c^	*PBSUR*^d^	*PBCAL*^e^	*T*Δ*S*^f^	Δ*G*_MM/PBSA_^g^
**WT_hACE2**	-708.99 ± 50.95	-94.94 ± 6.98	39.62 ± 15.64	-12.70 ± 1.81	748.62 ± 49.23	-56.77 ± 19.20	-11.24
**Delta_hACE2**	-2208.63 ± 98.11	-82.95 ± 6.94	4.32 ± 12.81	-11.24 ± 0.61	2212.95 ± 96.79	-72.56 ± 15.90	-17.31
**Mu_hACE2**	-2362.66 ± 65.03	-137.72 ± 7.46	37.73 ± 11.31	-16.77 ± 1.03	2400.40 ± 61.56	-57.64 ± 8.78	-59.12
**Omicron_hACE2**	-3481.08 ± 100.28	-88.56 ± 2.75	-7.07 ± 8,70	-11.38 ± 0.33	3411.01 ± 101.24	-51.79 ± 17.75	-55.22
**WT_TMPRSS2**	-324.64 ± 82.21	-70.29 ± 11.95	7.33 ± 14.28	-11.57 ± 2.21	331.97 ± 74.76	-61.01 ± 4.31	-13.52
**Delta_TMPRSS2**	-150.31 ± 64.66	-87.75 ± 8.12	20.64 ± 24.44	-13.63 ± 0.97	170.95 ± 47.12	-63.17 ± 5.01	-17.57
**Mu_TMPRSS2**	-225.74 ± 64.96	-149.42 ± 17.99	83.42 ± 25.38	-19.06 ± 2.02	261.37 ± 63.17	-58.19 ± 5.77	-26.86
**Omicron_TMPRSS2**	-64.12 ± 62.47	-76.95 ± 5.54	17.07 ± 10.28	-11.83 ± 1.23	81.19 ± 53.97	-65.93 ± 0.84	-5.78

^a^ Electrostatic energy in vacuum

^b^ van der Waals energy in vacuum

^c^ the polar part of solvation free energy

^d^ the non-polar part of solvation free energy

^e^ reaction field energy

^f^ conformational entropy difference multiplied by absolute temperature

^g^ the binding free energy calculated with MM/PBSA method

After the recognition by hACE2, SARS-CoV-2 SP undergoes significant structural arrangement and cleavage by transmembrane protease serine 2 (TMPRSS2), leading to membrane fusion and entry into host cells [22]. As shown in Table 2, the binding free energy of WT SP with TMPRSS2 is -13.52 kcal·mol^−1^, which is consistent with the previous molecular simulation results by Hussain’s group [65]. Both Mu and Delta SP bind to TMPRSS2 stronger than WT with binding free energy of -26.86 and -17.57 kcal·mol^−1^, respectively. However, molecular recognition of Omicron to TMPRSS2 is slightly weak with binding free energy of only -5.78 kcal·mol^−1^, indicating low catalytic efficiency of TMPRSS2. In fact, poor ability of membrane fusion into host cells in the Omicron variant has been demonstrated by previous research [66]. In addition, TMPRSS2 is mainly distributed in the lungs rather than the upper respiratory tract, [67] which partly explains why Omicron patients have less infectious in the lungs and fewer symptoms.

Based on energy decomposition data of the WT, Delta, Mu, Omicron systems, HC analyses were performed to obtain key residues favoring the association of SP with hACE2/ TMPRSS2. As shown from Supplementary Fig. 7, Most of key residues belong to RBD2_DPs with the exception of L455 and G496, further supporting that its conformational change is closely related to hACE2 binding ability. Although Mu has the strongest binding affinity with hACE2, residues Y449, G496, Q498 don’t contribute to its binding; the main influencing factors include Q498, T500, Y501, Y505, especially the mutation N501Y. For the Omicron variant, K417N may reduce the binding affinity of hACE2, [68] but G496S, Q498R and N501Y greatly restore its recognition efficiency during evolution [69].

As for TMPRSS2 interactions, the key residues in WT were P809/ S810/P812/ L821/ D843/ K921, and these in Delta, Mu and Omicron were P809/ S810/ P812/ L821/ D843/ K921, P809/ S810/ P812/ L821, D808/ P809/ S810/ K811/ P812/ L821/ D843/ R847, respectively. It will be helpful for the design of SARS-CoV-2 inhibitors based on TMPRSS2 recognition mechanism. The heptad repeats 1 (HR1) of SP plays a decisive role in transmembrane processes of SARS-CoV-2, and the higher the helicity of HR1, the stronger the fusion ability. As shown from Supplementary Fig. 8, the helicity of the WT, Delta, Mu and Omicron systems has little difference, maintaining at around 75%. In comparison, the helicity of Mu is slightly higher. It is speculated that antiviral peptides [70] targeting HR1 may have better therapeutic effects for Mu than for the other three systems. But according to Xie’s experiment, [71] Mu was less capable of fusing cells than Delta. Thus, the actual membrane fusion activity is complex and may be influenced not only by helicity but also by other participants in the fusion pathway. Zhu et al. [97] also found HR1 mutations might play a crucial role in enhancing fusion capacity. The Omicron variant is known to own many mutations in HR1, while the actual membrane fusion experimental data have not been published.

### Recommendations for therapeutic antibodies selection and modification

Based on the above analysis, the structural differences between SP WT and the Delta, Mu and Omicron variants were comprehensively compared (see Table 3), providing guidance for the selection and design of subsequent antibodies and vaccines.

**Table 3:** Comprehensive comparison of SP WT with three variants ^a^

Items	WT	Delta	Mu	Omicron
Structural stability	0	0	0	+
Conformational changes of epitopes	0	+	+ + +	+ +
Up/Down conformation	0	+ +	+	+
Binding affinity to hACE2	0	+	+ + +	+ +
Binding affinity to TMPRSS2	0	+	+ + +	-
Helicity	0	-	+	- -

^a^ In the comprehensive comparison, SP WT was taken as the control, and all the reference values were set to zero. The six signs (i.e., +  +  + , +  + , + , 0, -, –, –-) respectively represent significant-/moderate-/ low-improvement, similar, as well as low-/moderate-/significant-decrease

In order to treat COVID-19 more effectively, it is also necessary to summarize the escape of the SARS-CoV-2 dominant variant strains from existing therapeutic antibodies. Molecular simulation data showed that the Mu variant may possess the most resistance to NTD- and RBD2-targeted antibodies, while the Delta and Omicron variants respectively to RBD1- and RBD2-targeted antibodies. In the subsequent recommendation and modification of therapeutic antibodies, three suggestions were proposed: (1) NTD-targeted antibodies with small volume and small SASA are recommended, while RBD2-targeted antibodies having small volume and large SASA may be of more clinical value; (2) RBD1-targeted antibodies aren’t recommended for the Delta variant because of its strong steric hindrance; (3) therapeutic antibodies with positive ESP is recommended, in response to the tendency of alkaline mutations in SP.

### Vaccine effectiveness evaluation and design

According to a serological experiment, SP RBD is the core target of ~ 90% plasma or serum neutralizing antibodies from almost 650 infected individuals by SARS-CoV-2 [72]. The mutation-induced conformational changes in RBD may influence the immunogenicity of SPs, resulting in partial vaccine failure [73]. Based on our initial analyses, Mu and Omicron with bigger RBD conformational differences from WT may escape from neutralizing antibodies elicited by infection or vaccination. These newly emerging variants (e.g., Delta, Mu and Omicron) have shown different degrees of neutralization resistance to antibodies elicited by three vaccines currently under Emergency Use Authorization (EUA). Specifically, mRNA-1273 (Moderna), BNT162b2 (Pfizer-BioNTech) and Ad26.CoV2.S (Johnson) respectively increased neutralizing titers by 2.2-3.4, 2.1-8.7, 22.8-38 fold against Delta, Mu and Omicron over WT, [8, 74–77] showing stronger immune escape. Similarly, other SARS-CoV-2 vaccines also showed reduced neutralization to variant strains, i.e., AZD1222 (Oxford/ Astra Zeneca), [78] Covishield (Serum Institute of India) [79], BBIBP-CorV (Sinopharm) [80] and CoronaVac (Sinovac) [81]. In fact, vaccine effectiveness depends on multiple factors, and its durability remains to be continuously monitored. Three mutations in SP (i.e., E484K, N501Y and D614G) affect the binding of serum neutralizing antibodies, all of which exist in Mu, [51] while only D614G is present in Delta. Similarly, the mutations K417N, S375F, G446S, S477N and E484A also explain why Omicron has strong resistance to vaccine-induced antibodies.

With comparative analyses of conformational changes in DPs, RBD2-targeted subunit vaccine can effectively cope with the Delta, Mu and Omicron variants—the three most prevalent SARS-CoV-2 variants strains. Since the structures of Delta’s RBD2, Mu’s NTD, Omicron’s NTD and RBD2 undergo significant change, vaccine-induced antibodies have developed resistance to the corresponding regions. The structural characteristics (see Table 3) of SP mutants should be taken into consideration in the design of personalized vaccine: (1) for Delta, significantly decreased volume and moderately decreased SA in NTD_DP, as well as abnormally increased volume and moderately increased SA in RBD2_DP; (2) for Mu, moderately decreased volume and significantly decreased SA in NTD_DP, as well as significantly decreased volume and moderately increased SA in RBD2_DP; (3) for Omicron, abnormally increased volume and moderately decreased SA in NTD_DP, as well as significantly decreased volume and increased SA in RBD2_DP. Given the large structural flexibility changes and key identification roles, attention should also be paid to the following residues in new-generation vaccine design, including N448/ Y495 in Delta, Y144S/ H146/ A475/ E484K/ N487/ Q498/ T500/ N501Y in Mu, R403/ D405/ K417N/ S375F/ G446S/ S477N/ E484A/ N501Y/ Y505H in Omicron.

In addition to vaccines and therapeutic antibodies, more diverse and effective strategies are being sought to deal with SARS-CoV-2 infection. For example, novel antiviral drugs have been continuously in development, such as oral drug Paxlovid (PF-07321332), [82] molnupiravir [83], and remdesivir [84], ect.

## Discussion

As SARS-CoV-2 continues to mutate and spread under natural selection pressure, the global COVID-19 pandemic shows no sign of ending. Like the law of jungle, the dominant virus variants are usually more infectious or better adapted to human immune system. The Delta variant was first detected in India in October 2020, and then quickly swept the globe to become the dominant COVID-19 variant strains. The Mu variant was then identified in Colombia in January 2021, which shows unexpectedly enhanced immune resistance to neutralization antibodies. Recently, the Omicron variant with more SP mutations has emerged as the most worrisome subtype, exhibiting stronger immune escape though the characteristics associated with disease progression are somewhat diminished. Based on evolution path of the virus, SARS-CoV-2 has gained a tacit agreement between infectivity and immunity escape for better adaptation to host environment.

In this work, comparative 100 ns MD simulations were respectively performed for twelve SARS-CoV-2 SP trimers (i.e., WT, Delta, Mu, Omicron, and their complexes respectively with hACE2 and TMPRSS2) to explore conformational differences of CPs and DPs. The computational results show that the Delta, Mu and Omicron variants have exhibited greater global and local conformational changes than WT, and possessed obvious inter-domain motion between RBD and S2 domains. By calculating RMSF, SASA, SA and volume parameters, it was found that the values in NTD region of SP mutants were all reduced, which was not conducive to binding with NTD-targeted antibody and resulted in immune escape. Nevertheless, the corresponding value of RBD2 increases, which helps to form a complex with hACE2 and facilitate virus transmission. In the Omicron variant with 37 mutations, RBD2_DPs is more involved in intramolecular H-bonds, while NTD_DPs has small but deep pocket, showing a new antibody resistance mechanism. Combined with viral infectivity and antibody escape, this work will provide some theoretical guidance for selection and optimization of subsequent therapeutic antibodies as well as vaccine design. Given the severity of the pandemic, there is a need to continue not only developing personalized vaccines against specific mutations, but also maintaining routine preventive measures such as social distancing, wearing masks and washing hands.

## Materials and methods

### System preparation

The initial SP structure was taken from the RCSB protein data bank (PDB), with all missing residues completed by the SWISS-MODEL server [85]. Considering that the active conformation of SP is RBD-up state, the PDB ID adopted by wild type (WT) and Delta variants are 7KJ2 [86] and 7V7S [87], respectively. Both crystal structures are analyzed by cryo-electron microscopy which have been a more powerful tool for high-resolution structure analysis of biological macromolecules than X-ray technology [88]. Taking the two as templates, Pymol mutagenesis tools and HadDock 2.4 sever [89 , 90] both were used to build the following twelve systems: (1) four full-length SP trimers including WT, Delta, Mu and Omicron variants; (2) four SP trimer complexes with hACE2 receptor; (3) four SP trimer docked complex models with TMPRSS2 (PDB ID: 7MEQ) [91, 92] For convenience of analysis, the eight SP complex systems are denoted by WT_hACE2/ TMPRSS2, Delta_hACE2/TMPRSS2, Mu_hACE2/ TMPRSS2 and Omicron_hACE2/ TMPRSS2, respectively.

### Molecular dynamics simulation

Molecular dynamics (MD) simulation is one of the most important techniques to study the thermodynamic and kinetic characteristics of biological macromolecules at atomic level. In this work, comparative MD simulations were performed for the twelve SP trimer systems (i.e., WT, Delta, Mu, Omicron and their complexes with hACE2 and TMPRSS2) using ff14SB force field and Amber 19 package [93 , 94]. The solutes were placed in octahedral box with a boundary of 15.0 Å, where solvent effect was characterized by TIP3P water model [95]. In addition to the temperature setting at 300 K for conventional MD simulations, both 310 and 320 K options have been added to assess whether temperature affects SP conformation.

Before MD simulation, two steps of energy optimization was carried out: (1) under the condition of solute-constraint with force constant of 500 kcal·mol^−1^·Å^−2^, the steepest descent optimization was performed for 5000 steps, followed by another 5000 steps of conjugate gradient minimization; (2) the solute-unrestrained minimization was also composed of the same steps above; the convergence criterion is that the energy gradient is less than 0.01 kcal·mol^−1^·Å^−2^. Finally, a two-stage 100 ns MD simulation was carried out after energy minimization. The first stage was the 5 ns solute-constrained dynamics with force constant of 10 kcal·mol^−1^ Å^−2^; the second was 95 ns unconstrained productive simulations, in which the SHAKE algorithm [96] was adopted to prevent the destruction of chemical bonds involving non-hydrogen atoms. During the whole MD simulation, the radius of non-bonded interaction was 10 Å; the integral time step was set to 2 fs; the conformational snapshots were sampled every 1 ps, thus total 100,000 conformations were collected for further statistical analysis; the motion process of the system was monitored with VMD 1.9.3 package [97].

### Prediction of antigen epitopes

Total 60 SP complexes (See Supplementary Table 2) with various antibodies were collected in RCSB PDB. Their interface residues were obtained using LigPlot + v2.2.4 software, [98] all of which might be potential antigen epitopes. According to residue's spatial characteristics of antigen epitopes, they can be divided into CPs and DPs. the former is composed of continuous linear amino acids, while the latter consists of spatially close residues that form a specific conformation. To predict two types of B-Cell epitopes, seven SP trimer sequences (i.e., WT, Alpha, Beta, Gamma, Delta, Mu, Omicron) were extracted from outbreak.info, and their spatial conformations were built based on 7KJ2 PDB template. These seven variants belong to the mainstream lineage, covering at least 75% of current SP sequence and more than 90% of infected population.

The CPs of SARS-CoV-2 SP were predicted by BepiPred-2.0 server [99] in IEDB with a threshold of 0.55 (corresponding specificity over than 0.817 and sensitivity less than 0.292). The DPs were predicted via the EPCES server tool [100] based on the chain B structure of SP (PDB ID: 7KJ2). It is evaluated using Consensus Scoring (EPCES) made up of six different scoring functions—residue epitope propensity, conservation score, sidechain energy score, contact number, surface planarity score and secondary structure composition.

### Free energy landscape

According to value minimum and barrier in free energy landscapes (FEL), the representative conformation and its transition can be obtained. The free-energy surface with the minimum value characterizes the maximum possible motion range of the system. By comparing demarcation point and free energy barrier, the conformational transition process can be monitored. As a common dimension reduction algorithm in data mining, principal component analysis (PCA) is widely used to describe the most important global functional motion for biomacromolecular systems [101]. In FEL analysis, the reduced dimensions can be constructed by PCA; The first (PC1) and second principal component (PC2) both serve as reaction coordinates for the mapping of free energy surface diagram. The calculation of conformational free energy is defined as follows:

1 $$\Delta G(\mathrm X)=-k_BT\ln P(\mathrm X)$$

where the reaction coordinate X denotes PC1 and PC2; *P* (X) is the probability of conformational distribution, representing the contribution of a particular conformation to the overall PCs; *k*_B_ and *T* express Boltzmann constant and absolute temperature in Kelvin, respectively.

### Conformational cluster Analysis

Based on the 100,000 snapshots obtained from MD simulation of the four systems (i.e., WT, Delta, Mu and Omicron variant), conformational cluster was performed using MMTSB packages [102]. The basic idea of cluster analysis is to calculate root mean square deviation (RMSD) values of Cα atoms between various conformations and to establish N × N RMSD matrix, where N is the number of snapshots. Assuming a RMSD threshold, if the RMSDs between two arbitrary conformations are smaller than this value, they are grouped into one certain cluster and removed out of cluster pool. Then, the above procedure is repeated for the remaining structures in cluster pool until all the conformations are grouped into a particular cluster. Conventionally, the snapshots with lowest energy in clusters are considered to be representative conformations. In this work, RMSD threshold was set as 3.0 Å, which is also the conformation amplitude range that MD refining can achieve.

### Antibody recognition interface

The main driving forces of antibody-antigen recognition include hydrophobic effect and electrostatic interactions, which can be partially evaluated by the changes in solvent accessible surface area (SASA), volume, electrostatic potential and pKa values. The SASA of macromolecules can be computed from MD trajectories with gmx sasa module using the algorithms of Eisenhaber et al. [103] In the four SP trimmers (i.e., WT, Delta, Mu and Omicron variant), the changes in both total and average surface area (SA) of each residue throughout MD trajectory over time were calculated. The pocket volumes was calculated with Discovery Studio v19.1 (Accelrys, San Diego, California, USA). The SASA and pocket volume both can be used to evaluate the recognition interface and binding strength of DPs to antibodies. The surface electrostatic potential was calculated and displaced with Adaptive Poisson Boltzmann Solver [104] (APBS) program embedded in Pymol 2.3.1 software. Based on the convergent 3D structures of the four SP trimmers, the pKa values of ionizable groups were predicted with PROPKA 3 package, [105] which was helpful to describe the electrical environment at the corresponding position.

### Evolutionary conservation

The sequence conservation of protein key residues is the basis for the evolution of viruses including SARS-CoV-2. In general, if a highly conserved region mutates, the virus may develop into a harmonious co-existence with the host. Conversely, it may simply be associated with the probability of natural mutation. In this work, the ConSurf server [106] was used to predict conservation degree of each amino acidic position; the full-length sequence of SARS-CoV-2 SP (PDB ID: 7KJ2) was used as template for the alignment. Based on the alignment of 150 sequences with a similarity between 90 and 35%, the conservation score was obtained on a scale from 1 (not conserved) to 9 (highly conserved).

### Binding free energy calculation and Energy decomposition

The average binding free energies of four SP trimers (i.e., WT, Delta, Mu and Omicron variant) with hACE2 and TMPRSS2 were computed by Molecular Mechanics/Poisson Boltzmann (MM/PBSA) method. Total 20 snapshots were collected from each MD trajectory every 5 ns intervals from 1 to 100 ns. The formula is as:

2 $$\Delta G_{bind}=\Delta H-T\Delta S=(\Delta E_{VDW}+\Delta E_{ELE}+\Delta G_{PBELE}+\Delta G_{PBSUR})-T\Delta S$$

where ∆*E*_VDW_ indicates intramolecular VDW energy under vacuum, while ∆*E*_ELE_ refers to the electrostatic fraction. ∆*G*_PBELE_ and ∆*G*_PBSUR_ represent the hydrophilic and hydrophobic part of solvation binding free energy, respectively. ∆*H* corresponds to total enthalpy change, and *T*∆*S* is the product of absolute temperature and conformational entropy change.

Energy decomposition analysis was performed with Molecular Mechanics/Generalized Born Surface Area (MM/GBSA) method [107]. The basic idea is that: (1) energy contribution of each residue can be divided into internal energy in vacuum, polar solvation energy calculated by Generalized Born (GB) model [108], non-polar solvation energy calculated by the LCPO algorithm [109]; (2) all sorts of energies can be decomposed to atoms in backbone and side-chain of each residue. In addition, internal energy in vacuum can also be divided into two parts, polar electrostatic and non-polar van der Waals interactions. According to LCPO algorithm, there is a positive correlation between non-polar solvation energy and SASA. Obviously, through energy decomposition, the key residues in the four SP trimer (i.e., WT, Delta, Mu and Omicron variant) recognizing hACE2 and TMPRSS2 can be obtained.

### Hierarchical clustering analysis

The identification of hot interaction spots of SP with hACE2 receptor and TMPRSS2 will aid the detection of antigen epitopes. In this work, key residues were identified by Hierarchical clustering (HC) analysis based on energy contributions per amino acid; R statistical analysis package is adopted in HC calculation, and Manhattan distance is used to describe the similarity levels among vectors. Manhattan distance is defined as: [110]

3 $$Distance \left(a,b\right)=\sum_{\mathrm{i}}({a}_{\mathrm{i}}-{b}_{\mathrm{i}})$$

where *i* indicates the dimensional index of individual binding energies *a* and *b* at residual level. To implement cluster discrimination, the Ward’s minimum variance method was adopted here. Finally, the calculation result files were processed using the online tree generator iTOL to formulate the hierarchical tree graph shown in color-coded modes [111].

## Supplementary Information

**Additional file 1: Supplementary Fig. 1** RMSD values of Cα atoms over time for **a **four full-length SP trimers and their **b **S2, **c** NTD, **d **RBD subdomains. **Supplementary Fig. 2** The** a** RMSD , **b** radius of gyration, **c** RMSF and **d** flexibility correlation for the WT, Delta, Mu and Omicron systems at different temperatures. **Supplementary Fig. 3** Superimposition of CPs representative conformations in the WT, Delta, Mu and Omicron systems. **Supplementary Fig. 4** Hydrogen bonds in the WT, Delta, Mu and Omicron systems. The second (i.e., Tot. H) and third (i.e., Frac.≥70%) columns respectively mean the total number of hydrogen bonds, as well as that with frequency over 70%. In the four systems, ^**a**^ the hydrogen bonds formed by DPs with the unique hydrogen bonds are shown in bold and marked with a pink frame. **Supplementary Fig. 5** The volume and surface area of **a** NTD-DP, **b** RBD1-DP and **c** RBD2-DP in the WT, Delta, Mu and Omicron systems over time. **Supplementary Fig. 6** Surface electrostatic potential and total pKa values of each DPs in the four systems. The color shifted from red to blue represents the charges from acidity to alkalinity. **Supplementary Fig. 7** Based on energy decomposition data of the WT, Delta, Mu, Omicron systems, HC analyses of key residues favoring the association of SP with hACE2/TMPRSS2. The most important cluster is colored in red, which can be considered to be the hot interaction spots. **Supplementary Fig. 8** The helicity of HR1 for the WT, Delta, Mu and Omicron systems. **Supplementary Table 1** Representative variant strains of SRAS-CoV-2. **Supplementary Table 2 **Total 60 SP complexes with various antibodies collected from RCSB PDB. **Supplementary Table 3 **Conservation of DPs residues of SARS-CoV-2 SP.

## Data Availability

The data is available with the corresponding author and will be provided upon the legitimate request.

## Abbreviations

AI: Artificial intelligence
CH: Central helix
CPs: Continuous epitopes
DPs: Discontinuous epitopes
ESP: Surface electrostatic potential
EUA: Emergency use Authorization
FDA: Food and drug administration
FEL: Free energy landscape
FP: Fusion peptide
hACE2: Human angiotensin converting enzyme-2
HR1: Heptad repeat 1
HR2: Heptad repeat 2
mAbs: Monoclonal antibodies
NTD: N-terminal domain
RBD: Receptor binding domain
RMSD: Radius of gyration root mean square deviation
RMSF: Root mean square fluctuation
SA: Surface area
SARS-CoV-2: Severe acute respiratory syndrome coronavirus 2
SASA: Solvent accessible surface area
SP: Spike protein
TMPRSS2: Transmembrane protease serine 2
VOC: Variant of concern
VOI: Variant of interest
WHO: World Health Organization
WT: Wild type
