# Supplementary material for: Omicron-included mutation-induced changes in epitopes of SARS-CoV-2 spike protein and effectiveness assessments of current antibodies
Source: Mol Biomed. 2022 Apr 24;3:12. doi: 10.1186/s43556-022-00074-3 (PMC9034971; doi:10.1186/s43556-022-00074-3)
Supplement: Supplementary file 1 — Additional file 1: Supplementary Fig. 1 RMSD values of Cα atoms over time for a four full-length SP trimers and their b S2, c NTD, d RBD subdomains. Supplementary Fig. 2 The a RMSD , b radius of gyration, c RMSF and d flexibility correlation for the WT, Delta, Mu and Omicron systems at different temperatures. Supplementary Fig. 3 Superimposition of CPs representative conformations in the WT, Delta, Mu and Omicron systems. Supplementary Fig. 4 Hydrogen bonds in the WT, Delta, Mu and Omicron systems. The second (i.e., Tot. H) and third (i.e., Frac.≥70%) columns respectively mean the total number of hydrogen bonds, as well as that with frequency over 70%. In the four systems, a the hydrogen bonds formed by DPs with the unique hydrogen bonds are shown in bold and marked with a pink frame. Supplementary Fig. 5 The volume and surface area of a NTD-DP, b RBD1-DP and c RBD2-DP in the WT, Delta, Mu and Omicron systems over time. Supplementary Fig. 6 Surface electrostatic potential and total pKa values of each DPs in the four systems. The color shifted from red to blue represents the charges from acidity to alkalinity. Supplementary Fig. 7 Based on energy decomposition data of the WT, Delta, Mu, Omicron systems, HC analyses of key residues favoring the association of SP with hACE2/TMPRSS2. The most important cluster is colored in red, which can be considered to be the hot interaction spots. Supplementary Fig. 8 The helicity of HR1 for the WT, Delta, Mu and Omicron systems. Supplementary Table 1 Representative variant strains of SRAS-CoV-2. Supplementary Table 2 Total 60 SP complexes with various antibodies collected from RCSB PDB. Supplementary Table 3 Conservation of DPs residues of SARS-CoV-2 SP. [file 43556_2022_74_MOESM1_ESM.docx]

**Supplementary information**

**Omicron-included Mutation-induced changes in epitopes of SARS-CoV-2 spike protein and effectiveness assessments of current antibodies**

**Du Guo^1, †^, Huaichuan Duan^2, †^, Yan Cheng^1^, Yueteng Wang^2^, Jianping Hu^2, *^, Hubing Shi^1, *^.**

^1^ Laboratory of Tumor Targeted and Immune Therapy, Clinical Research Center for Breast, State Key Laboratory of Biotherapy, West China Hospital, Sichuan University and Collaborative Innovation Center, Chengdu 610041, China

^2^ Key Laboratory of Medicinal and Edible Plants Resources Development of Sichuan Education Department, School of Pharmacy, Chengdu University, Chengdu 610106, China

* Corresponding author.

E-*mail addresses*: hjpcdu@163.com (J. Hu), shihb@scu.edu.cn (H. Shi)

^†^ These authors contributed equally to this work.


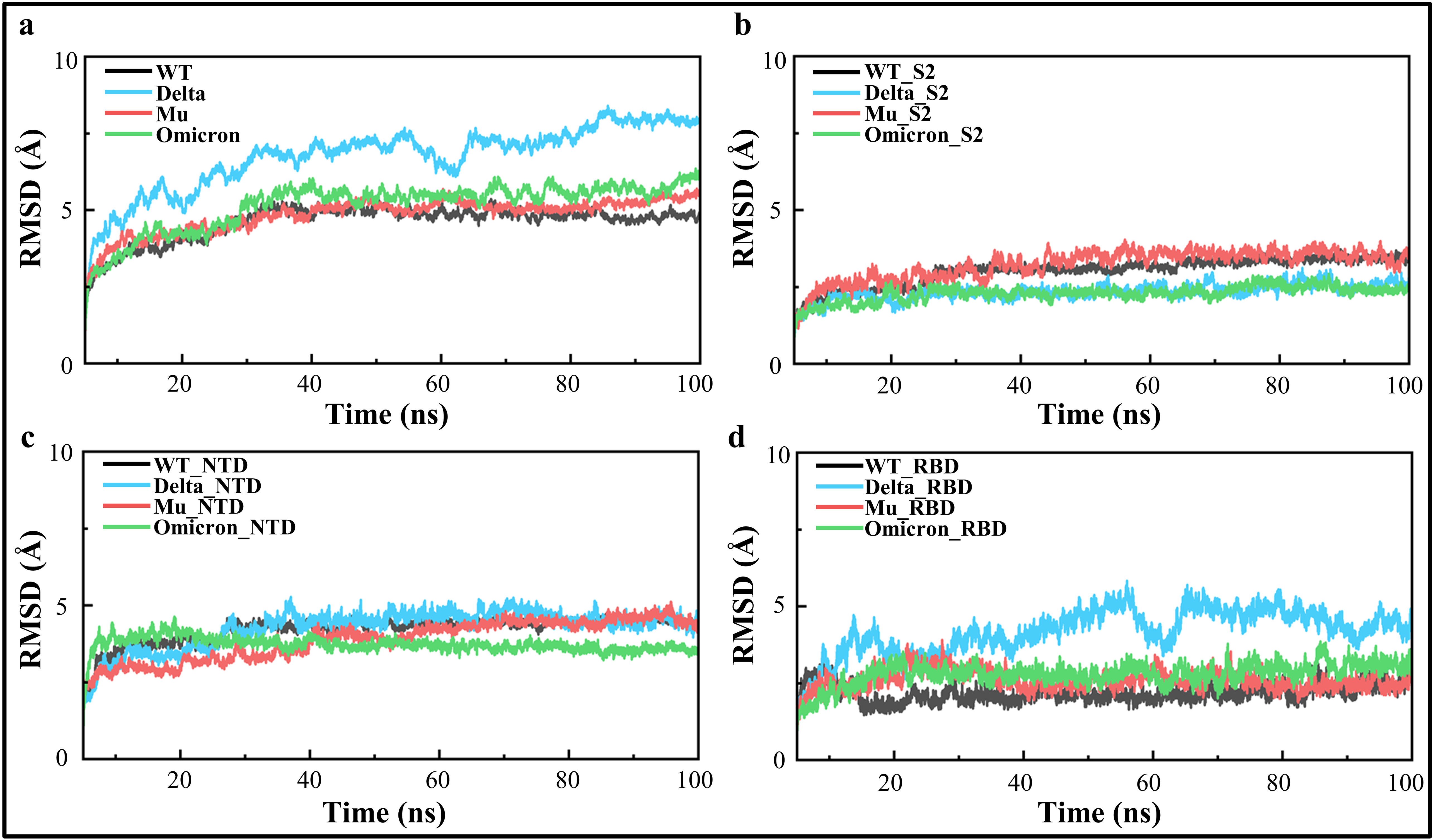


**Supplementary Fig. 1**


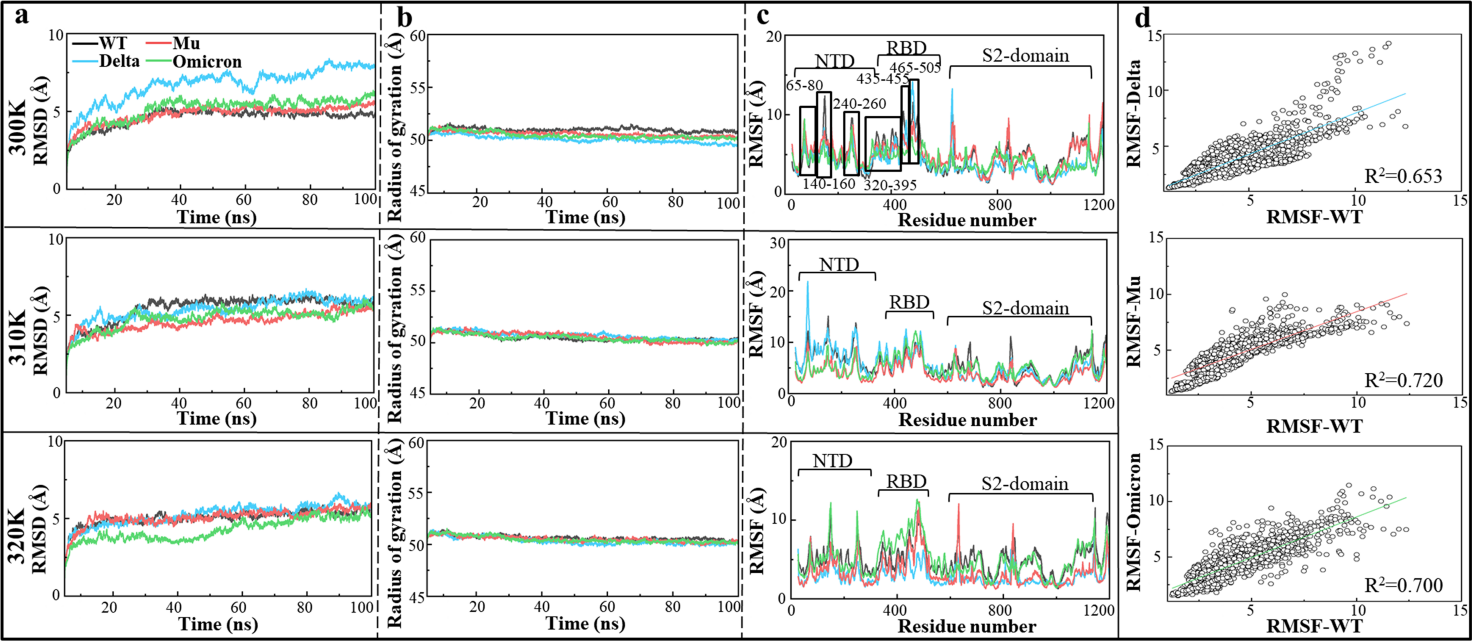


**Supplementary Fig. 2**


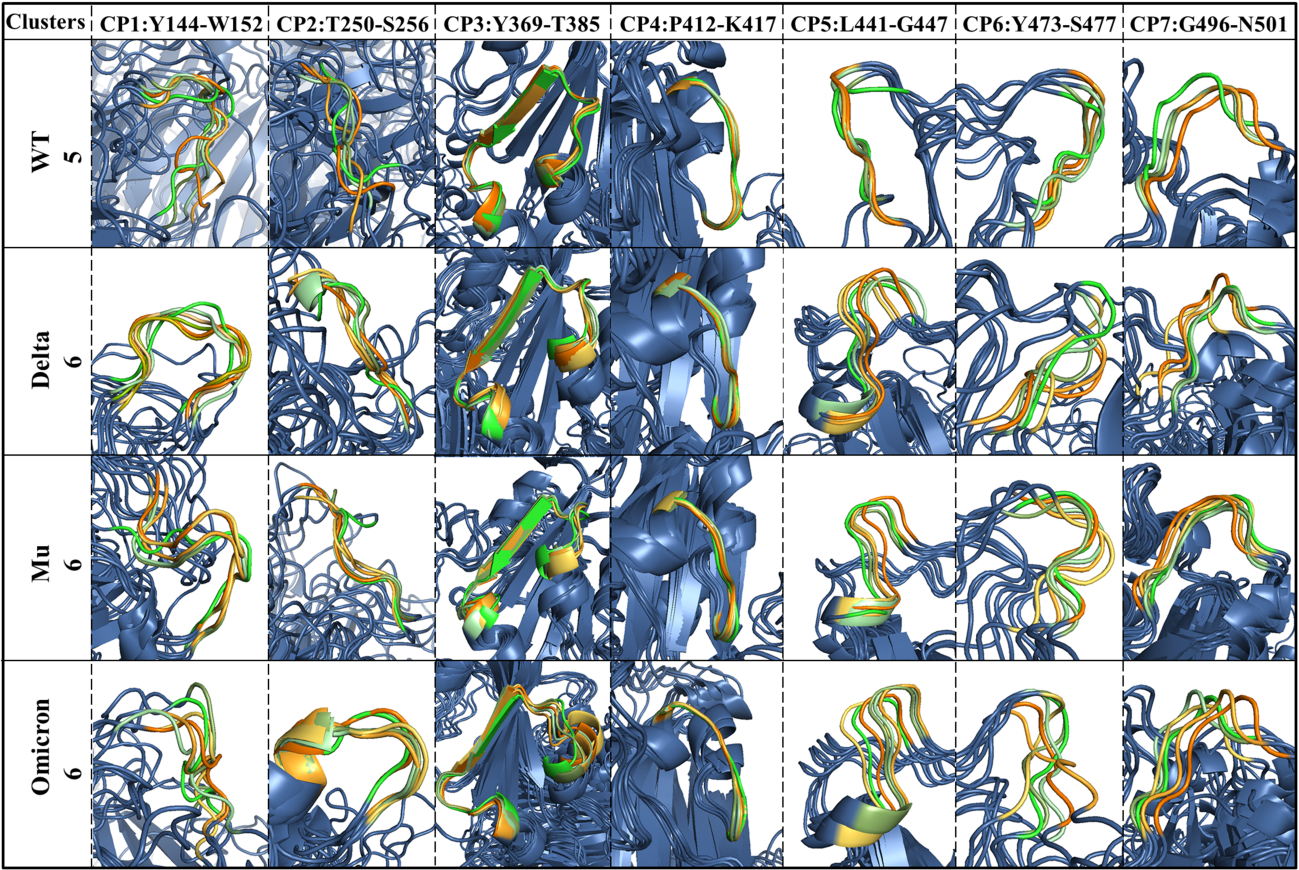


**Supplementary Fig. 3**


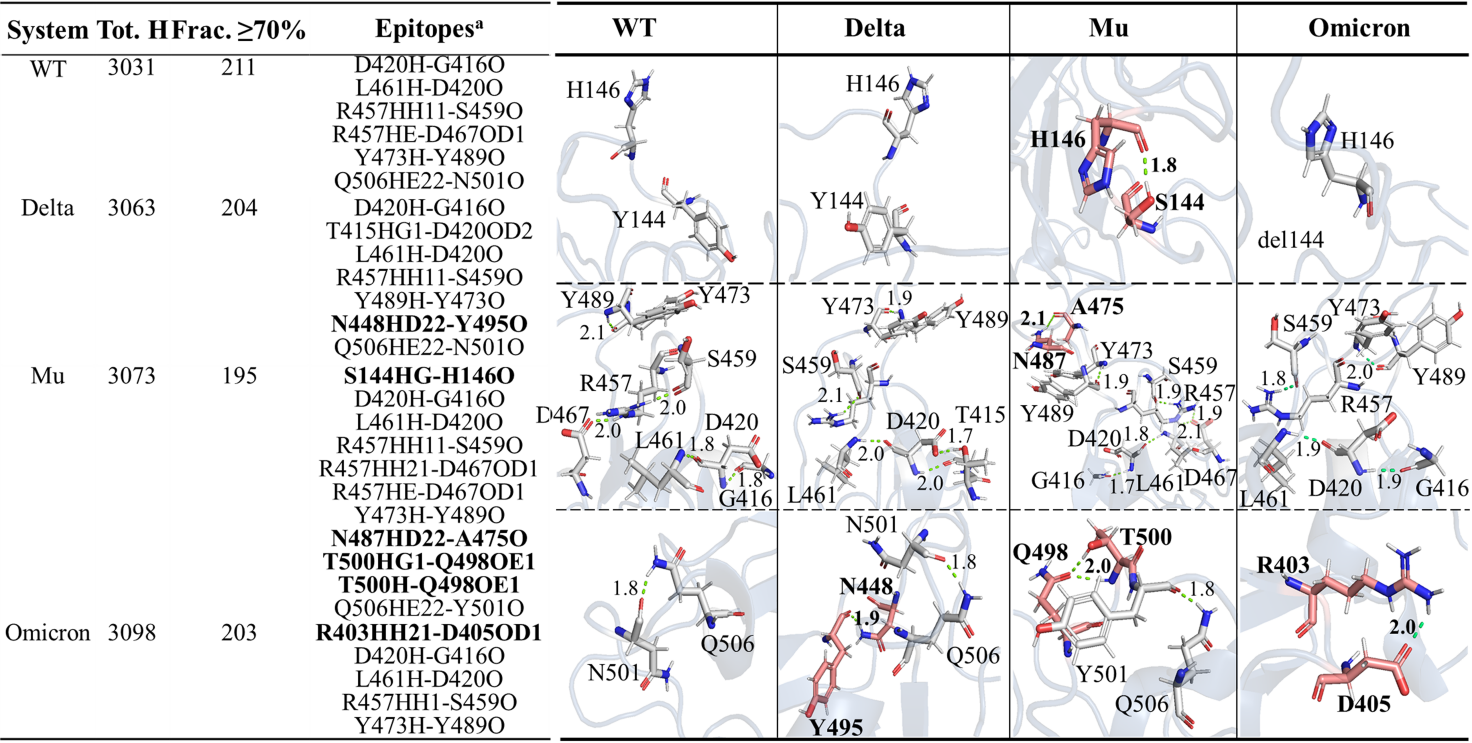


**Supplementary Fig. 4**


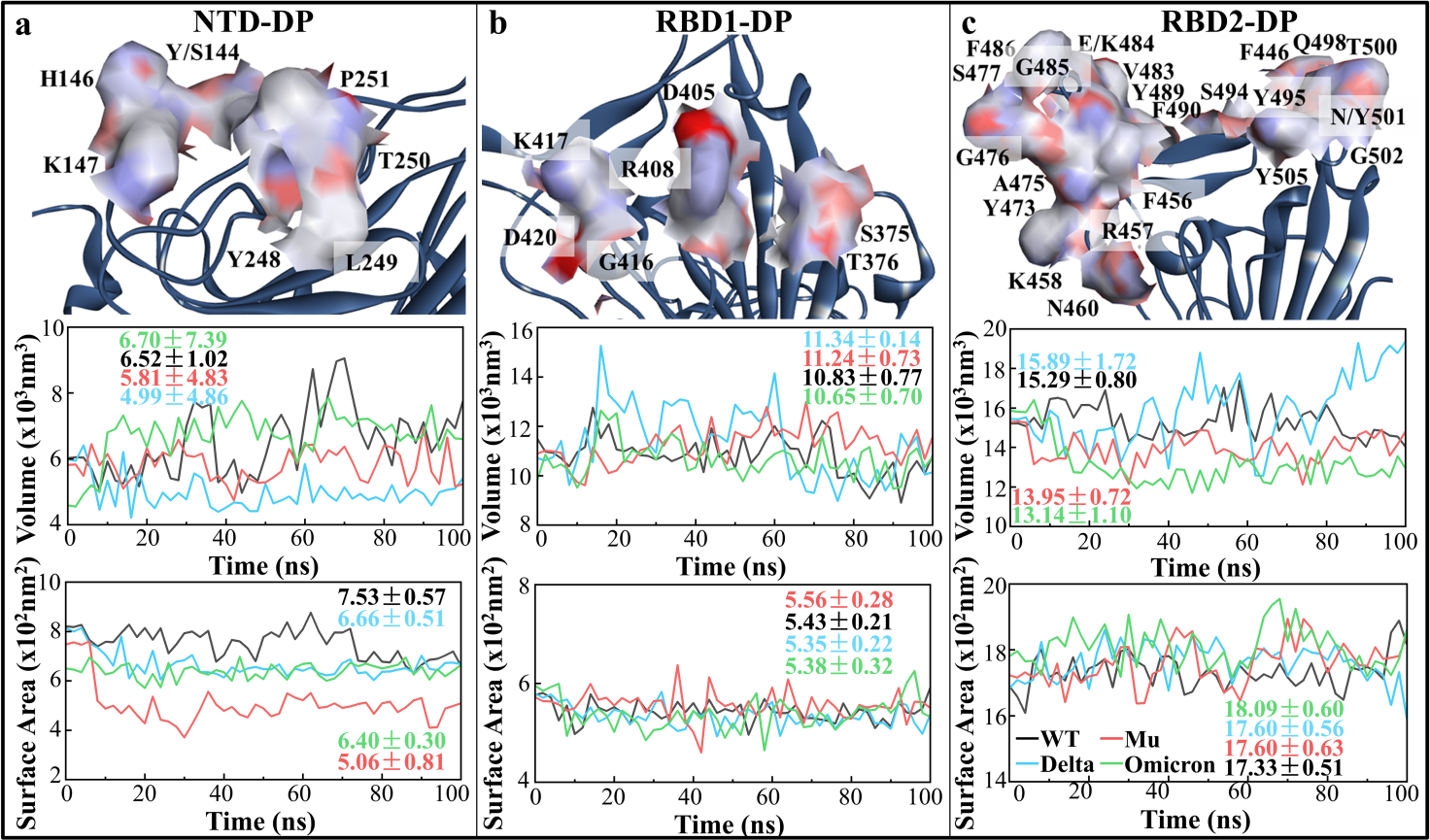


**Supplementary Fig. 5**


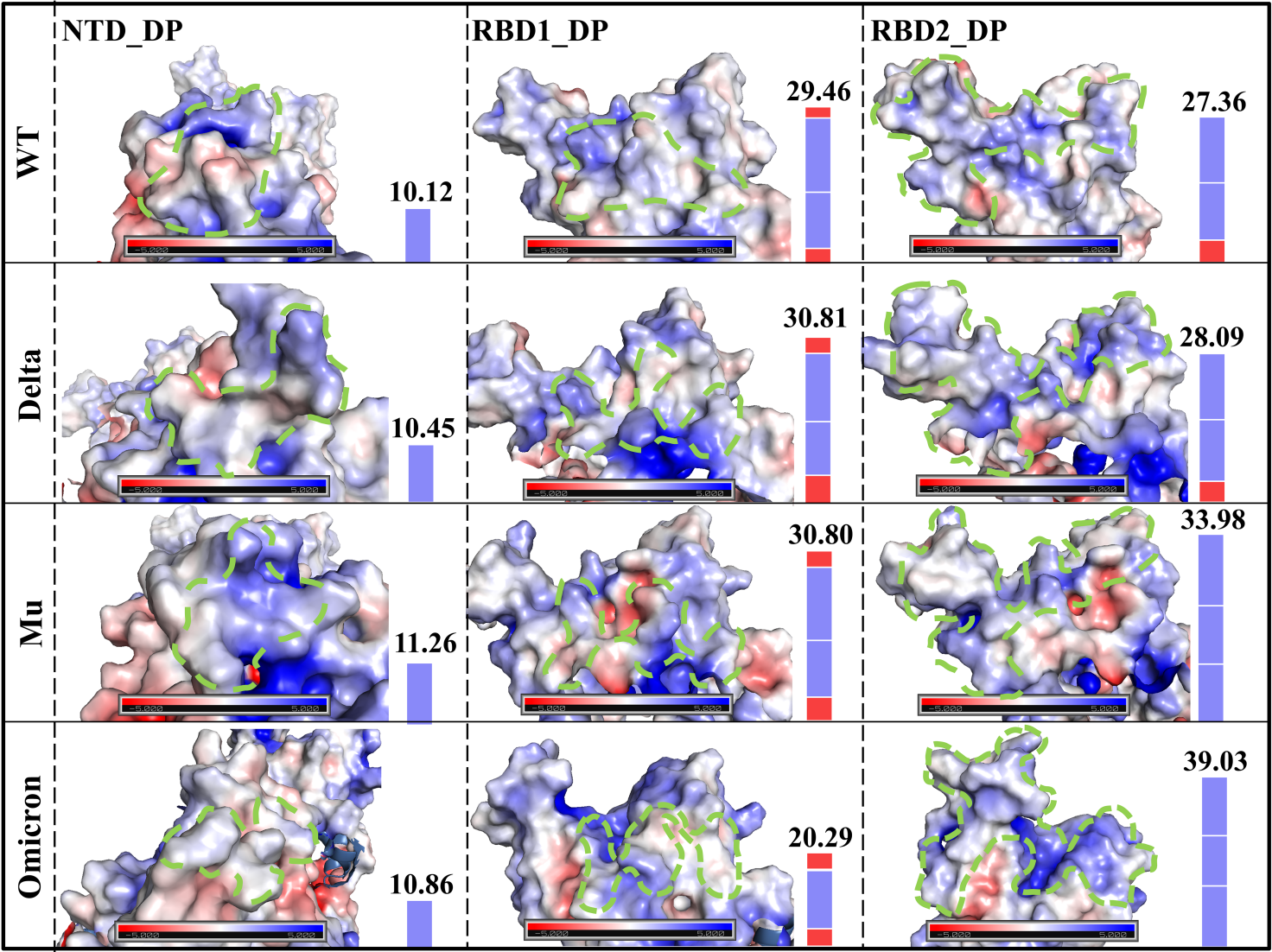


**Supplementary Fig. 6**


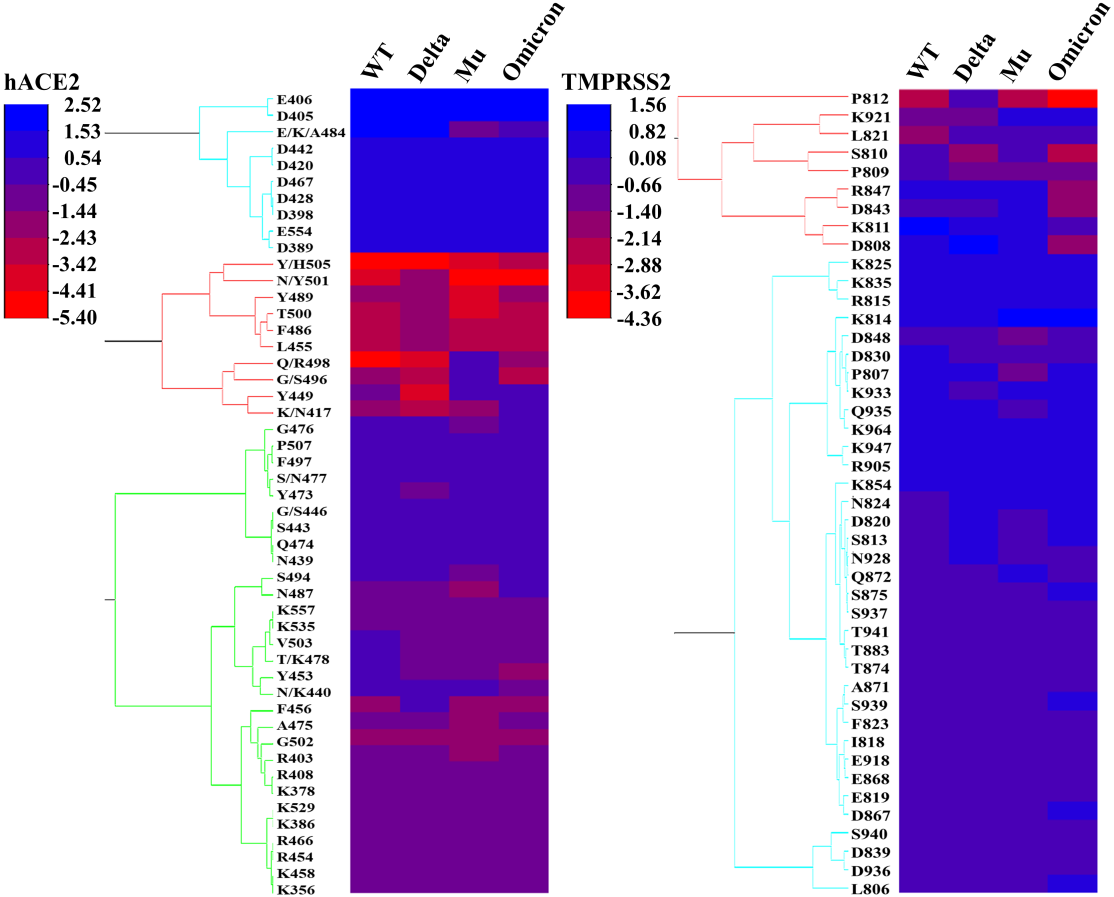


**Supplementary Fig. 7**


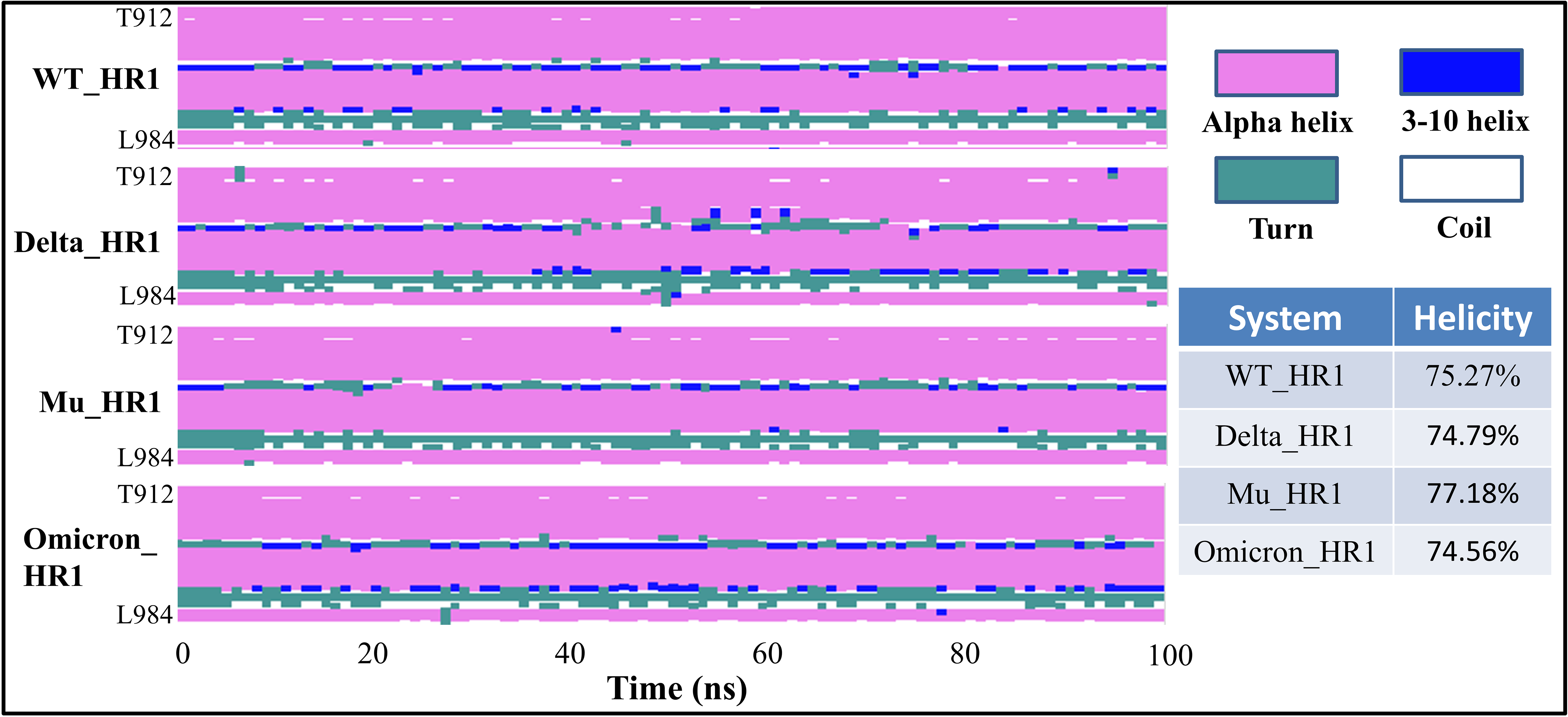


**Supplementary Fig. 8**

**Supplementary Table 1** Representative variant strains of SRAS-CoV-2

| **Definition** | **WHO Label** | **PANGO Lineage** | **Most common countries** | **Mutation sites** | **Earliest date** |
| --- | --- | --- | --- | --- | --- |
| VOC^a^ | Alpha | B.1.1.7 | United Kingdom 24.0%, United States of America 20.0%, Germany 10.0%, Sweden 6.0%, Denmark 6.0% | del69/70, del144/145, N501Y, A570D, D614G, P681H, T716I, S982A, D1118H | 7 Feb.  2020 |
|  | Beta | B.1.351 | United Kingdom 24.0%, United States of America 20.0%, Germany 10.0%, Sweden 6.0%, Denmark 6.0% | D80A, D215G, K417N, E484K, N501Y, D614G, A701V | 15 Feb.  2020 |
|  | Gamma | P.1 | United Kingdom 24.0%, United States of America 20.0%, Germany 10.0%, Sweden 6.0%, Denmark 6.0% | L18F, T20N, P26S, D138Y, R190S, K417T, E484K, N501Y, D614G, H655Y, T1027I, V1176F | Apr. 7  2020 |
|  | Delta | B.1.617.2 | United States of America 42.0%, India 13.0%, United Kingdom 11.0%, Germany 5.0%, Denmark 4.0% | T19R, E156G, del157/158, L452R, T478K, D614G, P681R, D950N | Sep. 22  2020 |
|  | Omicron | B.1.1.529 | United States of America 60.0%, Turkey 20.0%, Italy 3.0%, Germany 3.0%, India 2.0% | A67V, del69/70, T95I, G142D/del143-145, del 211/L212I, ins214EPE, G339D, S371L, S373P, S375F, K417N, N440K, G446S, S477N, T478K, E484A, Q493K, G496S, Q498R, N501Y, Y505H, T547K, D614G, H655Y, N679K, P681H, N764K, D796Y, N856K, Q954H, N969K, L981F | Nov. 15  2021 |
| VOI ^b^ | Mu | B.1.621 | United States of America 41.0%, Colombia 29.0%, Chile 9.0%, Spain 5.0%, Mexico 4.0% | T95I, Y144S, Y145N, R346K, E484K, N501Y, D614G, P681H, D950N | Oct. 14  2020 |

**VOC^a^** and **VOI^b^** are abbreviations for variant of concern and interest, respectively. Both have been associated with at least one of the following features: increased infectivity or detrimental change in COVID-19 epidemiology; increased irulence or change in clinical disease presentation; decreased effectiveness of public health, social measures, available diagnostics, vaccines, therapeutics.

**Supplementary Table 2** Total 60 SP complexes with various antibodies collected from RCSB PDB

| **Target** | **Year** | **PDB ID** | **Antibody** | **Binding site of Spike protein** |
| --- | --- | --- | --- | --- |
| RBD | 2020 | 6WPT | S309 | N334,L335,P337,G339,E340,N343,A344,T345,R346,K356,R357,S359,N360,L441,R509 |
|  |  | 6ZCZ | Nanobody H11-H4 | Y449,N450,L452,L455,F456,V483,E484,Y489,F490,L492,Q493,S494 |
|  |  | 6Z43 | Nanobody H11-D4 | Y449,N450,L452,F456,E484,Y489,F490,L492,Q493,S494 |
|  |  | 7BYR | Ab23-Fab | Y449,E484,G485F486,F490,Q493,S494,G496,Q498,Y505, |
|  |  | 6XEY | 2-4 | Y449,Y453,L455,F456,E484,G485,Y489,F490,L492,Q493,S494 |
|  |  | 7A5S | CR3022 | V42,F43,R44,S45,V47,L368,Y369,N370,F377,K378,G381,S383,P384,D428,T430,L390 |
|  |  | 7JV2 | S2H13 | G446,Y449,N481,G482,V483,E484,G485,F486,F490,S494 |
|  |  | 7JVA | S2A4 | Y369,N370,S371,A372,F374,S375,T376,F377,K378,C379,V382,S383,P384,T385,R408,  Q414 |
|  |  | 7KKK | Nanobody Nb6 | F342,N343,S371,S373,Trp436,N440,G446,Y449,L455,F456,E484,G485,F486,Y489,F490,Q493,S494,Y495,G496,Q498,N501, Y505 |
|  |  | 7DD2 | 3C1 | Y369,N370,A372,T376,F377,K378,C379,Y380,S383,P384,D405,V407,R408,Q409,Q414,  G502,V503,G504,Y505,Y508 |
|  | 2021 | 7KSG | Nanobody E | G446,G447,N448,N450,L452,F456,T470,E484,G485,F486,Y489,F490,L492,Q493,S494,  Q498 |
|  |  | 7KS9 | 910-30 | R403,T415,G416,K417,D420,Y421,L455,F456,R457,K458,N460,Y473,A475,G476,F486,  N487,Q493,S494,Y495,G496,Q498,T500,N501,G502,Y505 |
|  |  | 7CAC | H014 | Y369,A372,S373,F374,S375,T376,F377,K378,C379,Y380,P384,T385,K386,G404,D405,  R408,A411,P412,G413,Q414,N437,G504,Y508 |
|  |  | 7L0N | S304 | Y369,N370,F377,K378,C379,Y380,G381,V382,S383,P384,T385,K386,N388,L390,P412,  D428,F429 |
|  |  | 7BEL | COVOX-45 | R403,K417,Y453,L455,F456,E484,G485,F486,N487,C488,Y489,Q493,N501,G502,Y505 |
|  |  | 7BEN | COVOX-253 | L455,K458,Y473,A475,G476,S477,G485,F486,N487,Y489,Q493 |
|  |  | 7NEH | COVOX-269 | R403,D405,R408,T415,K417,D420,Y421,Y453,L455,F456,R457,K458,N460,Y473,Q474,A475,G476,F486,N487,Y489,Q493,Y495,Q498,T500,N501,G502,Y505 |
|  |  | 7BEK | COVOX-158 | R403,D405,E406,R408,Q409,T415,G416,K417,D420,Y421,L455,F456,R457,K458,N460,  Y473,Q474,A475,G476,S477,F486,N487,Y489,Y495,G496,Q498,T500,N501,G502,Y505 |
|  |  | 7ND3 | COVOX-40 | R403,T415,G416,K417,D420,Y421,L455,F456,R457,S459,N460,Y473,Q474,A475,G476,  S477,F486,N487,Y489,Y495,G496,T500,N501,G502,Y505 |
|  |  | 7ND4 | COVOX-88 | R403,K417,Y453,L455,F456,G485,F486,N487,Y489,Q493,T500,N501,G502,Y505 |
|  |  | 7ND5 | COVOX-150 | R403,D405,R408,T415,G416,K417,D420,Y421,Y453,L455,R457,K458,N460,Y473,A475,  G476,S477,F486,N487,Y489,Q493,Y495,Q498,T500,N501,G502,Y505 |
|  |  | 7ND7 | COVOX-316 | Y449,Y453,L455,F456,V483,E484,G485,F486,Y489,F490,L492,Q493,S494 |
|  |  | 7BEO | COVOX-253H55L | N440,L441,S443,K444,V445,G446,G447,N448,Y449,F490,L492,Q493,S494,Y495 |
|  |  | 7BEP | COVOX-384 | N334,L335,P337,G339,E340,N343,A344,T345,R346,A348,K356,R357,C361,L441 |
|  |  | 7NX8 | COVOX-222 | R403,D405,T415,G416,T417,D420,Y421,Y453,L455,F456,R457,K458,N460,Y473,Q474,  A475,G476,S477,F486,N487,Q493,Q498,T500,N501,G502,Y505 |
|  |  | 7L56 | 2-43 | Y449,L455,F456,V483,E484,G485,F486,Y489,F490,Q493,S494 |
|  |  | 7L57 | 2-15 | E484 |
|  |  | 7LXY | S2M11 | L441,K444,G446,L455,F456,Y449,E484,G485,F486,Y489,F490,L492,Q493,S494 |
|  |  | 7D0C | P5A-3A1 | T415,G416,K417,D420,Y421,L455,R457,K458,N460,Y473,Q474,A475,G476,S477,F486,  N487, G496,Y505 |
|  |  | 7D0B | P5A-3C12 | V483,E484,F486 |
|  |  | 7NX9 | EY6A | S366,Y369,N370,F377,C379,Y380,G381,V382,S383,P384,T385,K386,L390,F392,P412,  D427,D428,F429,L517 |
|  |  | 7M6H | BG7-20 | K417,K444,Y449,L452,L455,F456,I472,G482,V483,E484,G485,F486,N487,Y489,F490,  L492,Q493,S494,Q498,Y505 |
|  |  | 7MJH | VH ab8 | Y449,L455,F456,S477,V483,E484,G485,F486,N487,C488,Y489,F490,Q493,S494,Q498 |
|  |  | 7M71 | 5A6 | Y449,T470,E471,N481,G482,V483,E484,G485,F486,N487,Y489,F490,L492,Q493 |
|  |  | 7M6I | BG1-24 | Y351,Y449,L452,T470,I472,Y473,G482,V483,E484,G485,F486,Y489,F490,L492,Q493,  S494 |
|  |  | 7MJL | IgG ab1 | R403,T415,G416,K417,D420,Y421,Y453,L455,F456,R457,K458,N460,Y473,A475,G476,S477,F486,N487,Y489,Q493,Y501,Y505 |
|  |  | 7E8M | P2C-1F11 | R403,T415,G416,N417,D420,Y421,Y453,L455,F456,R457,K458,N460,Y473,A475,G476,  S477,F486,N487,Y489,Q493,T500,Y501,G502,Y505 |
|  |  | 7M6F | BG1-22 | T415,Y421,A475,G476,N487,S494,G502 |
|  |  | 7M6G | BG7-15 | N439,N440,S443,K444,V445,G446,G447,Y449,S494,N450,P499,T500,Q506 |
|  |  | 7MKL | SARS2-38 | N440,L441,K444,V445,Y449,T500 |
|  |  | 7M7B | 3D11 | Y369,N370,S371,A372,F374,S375,T376,K378,C379,S383,P384,T385,R408,Q414 |
|  |  | 7M6E | BG10-19 | G339,F342,N343,T345,R346,L368,S371,S373,F374,Trp436,S438,N440,L441,K444,N448 |
|  |  | 7N0G | Nanobody Sb45 | Y351,S375,T376,R403,D405,E406,E409,G413,Q414,D427,D428,G446,Y449,N450,L452,Y453,L455,F456,T470,E471,I472,G482,V483,E484,G485,F490,L492,Q493,S494,S501,V503,G504,Y505 |
|  |  | 7E8C | N9 | R403,D420,Y421,L455,F456,R457,K458,N460,Y473,A475,F486,N487,Q498,Q493,T500,  N501,G502,Y505 |
|  |  | 7E8C | 368-2 | Y449,N450,L452,I472,N481,G482,V483,E484,F490,L492 |
|  |  | 7CYP | HB27 | N437,N440,V445,G446,Y449,Q498,P499,T500,N501,G502,V503,Y505,Q506 |
|  |  | 7MY3 | Nanobody Nb12 | Y369,N370,A372,F374,S375,T376,K378,T385,G404,R408,N440,F484,Y489,V503,Y508 |
|  |  | 7MY2 | Nanobody Nb30 | N370,A372,F374,S375,T376,F377,K378,C379,Y380,G381,V382,P384,G404,D405,R408,  P412,Q414,N437,V503,G504,Y508 |
| NTD | 2020 | 7C2L | 4A8 | Y144,Y145,H146,K147,K150,Trp152,H245,R246,Y248,L249 |
|  | 2021 | 7L2D | 1-87 | Y144,Y145,H146,K147,K150,Trp152,H245,R246,Y248,L249,T250,P251,G252,S254,S255,S256 |
|  |  | 7L2E | 4-18 | Q14,C15,V16,N17,T19,G142,Y144,K147,E156,R158,L244,H245,R246,S247,Y248,L249,  T250,P251,G252,S256 |
|  |  | 7L2F | 5-24 | Q14,C15,Y144,K147,R158,E154,E156,R246,Y248,L249,T250,P251,D253 |
|  |  | 7NDC | COVOX-159 | Q14,Y144,H146,K147,F157,G252,D253 |
|  |  | 7LY0 | S2M28 | V16,N17,Y144,H146,K147,N148,S155,R158,R246,L249,T250,P251,G252,D253,S254 |
|  |  | 7LXX | S2L28 | V16,Y144,R246,S247,Y248,T250,P251,G252,D253,S254,S255,S256,G257 |
|  |  | 7LXW | S2X333 | V16,N17,F140,G142,V143,Y144,H146,K147,Trp152,E154,E156,R158,L244,H245,R246,  L249,P251 |
|  |  | 7NTC | P008_056 | S71,K97,T124,Y145,H146,K147,K150,S151,Trp152,E180,G181,N185,V213,H245,S247,  Y248,L249,T259,A260,A262 |
|  |  | 7E8C | N9 | Q14,Y144,H146,K147,E156,F157,R246,Y248,L249,T250,P251,G252,D253,S254,W258 |
|  |  | 7DZY | 2490 | G181,K182 |
|  |  | 7DZX | 8D2 | Q183 |

**Supplementary Table 3** Conservation of DPs residues of SARS-COV-2 SP

| **Subdomains** | **DPs** | **Conservation ^a^** | **Secondary structure^b^** |
| --- | --- | --- | --- |
| **NTD_DP** | Y144,H146,K147,Y248,L249 | 1 | Loop |
|  | T250 | 3 | Loop |
|  | P251 | 8 | Loop |
| **RBD1_DP** | D405,K417 | 1 | Helix |
|  | S375,T376 | 9 | Beta sheet |
|  | R408,D420 | 9 | Helix |
|  | G416 | 9 | Loop |
| **RBD2_DP** | G446,F456,K458,G476,S477,E484,G485,F486,F490,S494,Q498,T500,N501,G502 | 1 | Loop |
|  | V483 | 2 | Loop |
|  | Y473,Y489 | 3 | Loop |
|  | N460,A475 | 4 | Loop |
|  | Y505 | 5 | Helix |
|  | A457,Y495 | 9 | Loop |
| **S2** | P793 | 1 | Loop |
|  | K814 | 3 | Loop |

**^a^** The larger the score is, the higher the conservation is. When the values is less than 3, it is defined as weak conservative residues.

**^b^** Secondary structure of the region to which the residue belongs.
